# Excitons in mesoscopically reconstructed moiré heterostructures

**DOI:** 10.1038/s41565-023-01356-9

**Published:** 2023-03-27

**Authors:** Shen Zhao, Zhijie Li, Xin Huang, Anna Rupp, Jonas Göser, Ilia A. Vovk, Stanislav Yu. Kruchinin, Kenji Watanabe, Takashi Taniguchi, Ismail Bilgin, Anvar S. Baimuratov, Alexander Högele

**Affiliations:** 1 Fakultät für Physik, Munich Quantum Center, and Center for NanoScience (CeNS), Ludwig-Maximilians-Universität München, Munich, Germany; 2 PhysNano Department, ITMO University, Saint Petersburg, Russia; 3 Center for Computational Materials Sciences, Faculty of Physics, University of Vienna, Vienna, Austria; 4 Nuance Communications Austria GmbH, Vienna, Austria; 5 Research Center for Functional Materials, National Institute for Materials Science, Tsukuba, Japan; 6 International Center for Materials Nanoarchitectonics, National Institute for Materials Science, Tsukuba, Japan; 7 Munich Center for Quantum Science and Technology (MCQST), München, Germany; 8 Present Address: Beijing National Laboratory for Condensed Matter Physics, Institute of Physics, Chinese Academy of Sciences, Beijing, P. R. China; 9 Present Address: School of Physical Sciences, CAS Key Laboratory of Vacuum Physics, University of Chinese Academy of Sciences, Beijing, P. R. China

**Keywords:** Two-dimensional materials, Two-dimensional materials, Two-dimensional materials

## Abstract

Moiré effects in vertical stacks of two-dimensional crystals give rise to new quantum materials with rich transport and optical phenomena that originate from modulations of atomic registries within moiré supercells. Due to finite elasticity, however, the superlattices can transform from moiré-type to periodically reconstructed patterns. Here we expand the notion of such nanoscale lattice reconstruction to the mesoscopic scale of laterally extended samples and demonstrate rich consequences in optical studies of excitons in MoSe_2_–WSe_2_ heterostructures with parallel and antiparallel alignments. Our results provide a unified perspective on moiré excitons in near-commensurate semiconductor heterostructures with small twist angles by identifying domains with exciton properties of distinct effective dimensionality, and establish mesoscopic reconstruction as a compelling feature of real samples and devices with inherent finite size effects and disorder. Generalized to stacks of other two-dimensional materials, this notion of mesoscale domain formation with emergent topological defects and percolation networks will instructively expand the understanding of fundamental electronic, optical and magnetic properties of van der Waals heterostructures.

## Abstract (web summary)

Moiré lattice reconstruction on mesoscopic length scales gives rise to diverse exciton signatures within emergent domains of different dimensionality.

## Main

Vertical assemblies of twisted or lattice-mismatched heterobilayers of two-dimensionaltransition metal dichalcogenides (TMDs) with moiré-modulated interlayer coupling give rise to correlated Hubbard-model physics^1^—exhibiting signatures of collective phases in both transport^2–5^ and optical experiments^6–9^. Periodic moiré interference patterns have profound effects on the electronic band structure due to formation of flat mini-bands that enhance many-body correlations, and induce emergent magnetism^6^, correlated insulating states^2–4,7–9^ or Wigner crystals^7^. Moiré effects also result in rich optical signatures of intralayer^10^ and interlayer^11–14^ excitons that are formed by Coulomb attractions among layer-locked and -separated electrons and holes, with angle-controlled exciton valley coherence and dynamics^15,16^, optical nonlinearities^17^ or correlated excitonic insulating states^18^.

Despite the extensive optical studies of moiré effects in TMD heterobilayers such as MoSe_2_–WSe_2_ (ref. ^19^), a consolidated picture of the rich experimental features remains elusive^20^. The experimental results for the peak energies of the interlayer exciton photoluminescence (PL)^21,22^, *g*-factor^11,22^ and degree of polarization^11,12^ are inconsistent, and the PL spectra can differ substantially from spot to spot even in the same sample^23^. The diversity of models invoked to explain the plethora of experimental signatures is not inherent to the theory of moiré excitons^24–26^ but is instead related to variations in actual samples. For TMD bilayers with small twist angles, in particular, canonical moiré superlattices are known to transform into periodic domains of distinct atomic registries in triangular or hexagonal tiling^27–33^, as dictated by the competition between the intralayer strain and interlayer adhesion energies^34–36^. Correlative studies of reconstruction and exciton features are so far limited to twisted homobilayers^28,29^, providing only indirect insight into exciton landscapes of mesoscopically reconstructed moiré heterostructures.

### Canonical and periodically reconstructed moiré heterostructures

**Fig. 1:**
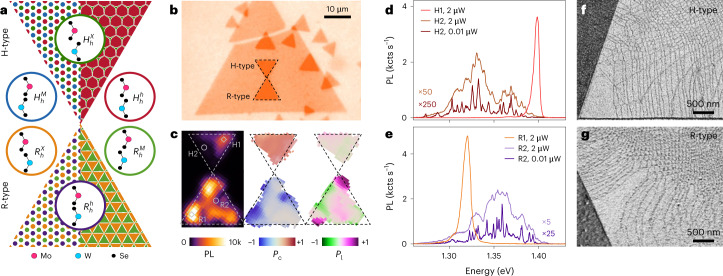
Characteristics of MoSe_2_–WSe_2_ HBLs in H- and R-type stacking. **a**, Schematics of H- and R-type heterostacks with ideal moiré (left) and periodically reconstructed (right) patterns. The coloured regions represent high-symmetry atomic registries, as illustrated in the respective circles. **b**, Optical micrograph of sample 1 with H- and R-stacks (delimited by dashed lines) of CVD-grown MoSe_2_ monolayers (small triangles) on a large WSe_2_ monolayer (large triangle). **c**, Interlayer exciton PL map (left) with selected bright (H1, R1) and dark (H2, R2) spots indicated by diamonds and circles, respectively, as well as *P*_c_ (middle) and *P*_l_ (right) maps for the H and R-stacks in **b**. **d**,**e**, Photoluminescence spectra at the bright and dark spots marked in **c**. At an excitation power of 2 μW, the H1 and R1 spectra are representative for regions with a single bright peak, whereas the H2 and R2 spectra (scaled by 50 and 5, respectively) are characteristics of dark regions with broad and structured PL, which evolves into narrow peaks at a low excitation power of 0.01 μW (scaled by 250 and 25, respectively). All spectroscopy data were recorded on sample 1. **f**,**g**, Scanning electron micrographs of H- (**f**) and R- (**g**) heterostacks recorded with secondary electron imaging. Source data

Ideal moiré heterostructures emerge in vertical heterobilayer (HBL) assemblies of distinct TMD monolayers without an inversion centre, and distinguish between parallel and antiparallel alignments near 0° and 180° twist angles of R- and H-stackings. Due to the lattice mismatch of the monolayer constituents, both the R- and H-type heterostacks form moiré patterns with superlattice constants of ~100 nm in aligned MoSe_2_–WSe_2_ HBLs, which reduces asymptotically to the monolayer lattice constant with increasing twist away from the high-symmetry configurations. Following lateral translation through a moiré supercell, each stacking modulates through points of highly symmetric atomic registries (${H}_{h}^{h}$, ${H}_{h}^{M}$, ${H}_{h}^{X}$ and ${R}_{h}^{h}$, ${R}_{h}^{M}$, ${R}_{h}^{X}$), which are shown schematically in the coloured circles of Fig. 1a for a heterostack of top MoSe_2_ and bottom WSe_2_ monolayers.

For rigid lattices, the resulting moiré pattern is shown on the left side of the H (top) and R (bottom) triangles (Fig. 1a). The positions of high-symmetry registries with identical areas are represented by their respective colours, spanning a periodic lattice of laterally alternating registries with gradual interconversion according to the geometrical interference condition. In the presence of local lattice deformation, however, the ideal moiré pattern transforms into periodically reconstructed patterns, which are shown on the right side of the triangles. Driven by competition between intralayer strain and interlayer adhesion energy, energetically favoured registries expand at the expense of unfavourable ones into periodic domains with areas inversely proportional to the squared twist angle *θ*^2^. Theoretical and experimental results^27,34^ indicate for H-type heterostacks that only ${H}_{h}^{h}$ stacking prevails in hexagonal domains after reconstruction, whereas in R-type heterostacks, ${R}_{h}^{X}$ and ${R}_{h}^{M}$ consolidate into tessellated triangular domains as two equally optimal registries.

The ideal and reconstructed moiré landscape scenarios of Fig. 1a can in principle be discerned by optical spectroscopy, despite the length-scale mismatch between the optical spot size (on the order of a micrometre) and the domain size (dimensions well below 100 nm). By using the distinct PL characteristics of interlayer excitons in atomic registries of MoSe_2_–WSe_2_ HBLs listed in Table 1, the observer could infer the contribution from each domain present in the optical spot. By virtue of unique spin-valley configurations, stacking symmetries and related degrees of interlayer coupling, R- and H-type interlayer excitons exhibit distinct transition energies^25,26,36–38^, oscillator strengths^37,38^ and dipolar selection rules^25,26,39^ that are accessible via optical spectroscopy. Moreover, magneto-luminescence experiments allow the assignment of interlayer exciton PL to the domains of specific registries using first-principles calculations of exciton Landé *g*-factors^38,40,41^ and experimental values. This conceptual clarity, however, is contrasted by the puzzling diversity of moiré exciton signatures in MoSe_2_–WSe_2_ HBLs.

**Table 1:** Theoretical parameters of interlayer excitons in distinct atomic registries of MoSe_2_–WSe_2_ HBLs

Stacking	Singlet				Triplet			
	Energy (eV)	∣μ∣ (Debye)	Polarization	*g*-factor	Energy (eV)	∣μ∣ (Debye)	Polarization	*g*-factor
${R}_{h}^{X}$ (optimal)	1.33	1.47	*σ*^−^	+5.8	1.35	0.70	*σ*^+^	–10.5
${R}_{h}^{h}$	1.38	2.06	*σ*^+^	–6.4	1.40	0.19	*z*	11.0
${R}_{h}^{M}$ (optimal)	1.48	1.09	*z*	6.3	1.50	0.02	*σ*^−^	+10.9
${H}_{h}^{M}$	1.39	0.40	*z*	13.1	1.37	0.10	*σ*^−^	+17.6
${H}_{h}^{X}$	1.41	0.69	*σ*^+^	–13.2	1.39	0.40	*z*	17.7
${H}_{h}^{h}$ (optimal)	1.42	2.14	*σ*^−^	+12.9	1.40	0.42	*σ*^+^	–17.6

The transition energy (estimated from ref. ^36^), oscillator strength (proportional to the square of the dipole moment ∣**μ**∣), polarization selection rule, and *g*-factor for zero-momentum *KK* or ${K}^{{\prime} }K$ interlayer excitons in R- and H-stacking in spin-singlet (electron and hole with antiparallel spin) and spin-triplet (electron and hole with parallel spin) configurations. Note that singlet (triplet) interlayer excitons are the lowest-energy states in R (H) stacking.

### Mesoscopic reconstruction in experiment and theory

In this work we fabricated HBL samples from MoSe_2_ and WSe_2_ monolayers exfoliated from native crystals or synthesized by chemical vapour deposition (CVD) (see the Methods for details). For the sample in Fig. 1b based on CVD-grown monolayers, we placed single-crystal MoSe_2_ triangles on top of a large WSe_2_ triangular monolayer by standard dry-transfer. The resulting HBL triangles with small-twist-angle H- and R-stackings are delimited by dashed lines in the optical micrograph of Fig. 1b. All of the HBLs were encapsulated in hexagonal boron nitride (hBN) to access narrow exciton linewidths in cryogenic spectroscopy^42^.

Figure 1c shows the PL characteristics in the spectral bandwidth of interlayer excitons. The laterally extended maps recorded at 3.2 K show the integrated PL intensity and the degrees of its circular (*P*_c_) and linear (*P*_l_) polarizations^43^. The PL map exhibits sizable intensity variations across both stacks, with much brighter emission in the R-stack. These variations are accompanied by changes in the spectral characteristics, shown representatively in Fig. 1d,e. In the upper-right and lower-left bright corners of the H- and R-type triangles (H1 and R1, respectively), the corresponding PL spectra feature only one peak at 1.40 and 1.33 eV, with the highest degrees of circular polarization and opposite signs in the *P*_c_ map of Fig. 1c. These signatures jointly suggest that both stacks feature areas on the scale of the optical spot that are entirely dominated by the respective triplet and singlet interlayer excitons of the ${H}_{h}^{h}$ and ${R}_{h}^{X}$ registries. Given the finite twist angle in our sample, the absence of PL contributions from all other registries is striking.

The signatures of bright spots are contrasted on positions with low PL (labels H2 and R2), and are shown as brown and purple spectra in Fig. 1d,e. At the expense of single-peak spectra, the PL is structured and spectrally dispersed over 100 meV on the low- and high-energy sides of the solitary peaks of the triplet ${H}_{h}^{h}$ and singlet ${R}_{h}^{X}$ interlayer excitons, respectively. On dark spots and under identical excitation conditions, the integrated PL is typically much lower (note the scaling by 50 and 5 for H- and R-type spectra, respectively), and *P*_c_ is reduced in its absolute value, although preserved in sign. These features of moiré effects^12,23,38^ evolve into a series of spectrally narrow peaks at reduced excitation powers, signifying interlayer exciton localization in moiré quantum dots^11,23^.

In addition to the co-existence of contrasting features in one sample, confusion arises from the observation of a finite degree of linear polarization with variations across the *P*_l_ map of Fig. 1c. According to Table 1, the dipolar selection rules of interlayer excitons dictate valley contrasting circularly polarized out-of-plane and in-pane *z*-polarized transitions^25,26,39^. Whereas the former constitute positive and negative *P*_c_ in the maps of the H- and R-type stacking (Fig. 1c), the latter should exhibit neither circular nor linear degrees of polarization when probed in back-scattering configuration^38^. By contrast, the map in Fig. 1c exhibits regions with high *P*_l_ (note the upper- and lower-left corners of the R-type triangle), reminiscent of quantum wire effects as in uniaxially strained moiré landscapes^44^ or HBL samples with transfer-induced layer corrugation^45^.

All of the above features manifest consistently across the samples of our studies (see Supplementary Notes 1 and 3 for other samples). The key to the understanding of such variations in the optical features on one sample is provided by mesoscopic reconstruction, which we visualize with secondary electron imaging in scanning electron microscopy (SEM)^29^. The images of H- and R-heterostacks near the triangle edges with stacking-sensitive contrast (Fig. 1f,g and Supplementary Note 1) provide evidence for the formation of large domains of one atomic registry separated by thin lines of domain walls. The domain networks observed in different samples exhibit common patterns on the mesoscopic scale: extended 2D domains at HBL tips and edges are surrounded by elongated 1D stripes that merge in the sample core into a network of finely structured domains with dimensions well below 100 nm, forming quasi 0D arrays.

Mesoscopic reconstruction is driven by the interplay of intralayer strain and interlayer adhesion energy. Recent theoretical work^34–36^ pointed out that moiré lattices of marginally twisted bilayers relax on the nanoscale into periodic domains by rearranging lattice atoms according to a vectorial 2D displacement field that overcompensates for the associated strain cost with the gain in interlayer adhesion. To provide intuition for domain formation on scales from a few to a few hundred nanometres, we adopt the theoretical model of lattice reconstruction^35^ and account for finite size effects and singular point rotations that can grow to large-area 2D domains of optimal stacking. In brief, we model the tip of a HBL with small twist angles by using an equilateral triangle, with its micrometre-sized base connecting to the rest of the triangle with ideal moiré periodicity (see Supplementary Note 2 for details). On the line bisecting the triangular tip into two equal halves, we place a point of zero-twist deformation to create an initial lattice-displacement field, which is modified in consecutive iterations to obtain a stacking configuration from the final displacement field, minimizing the sum of the intralayer strain and interlayer adhesion energies in the tip area. This procedure yields a series of reconstructed landscapes, characterized by their respective initial displacement fields.

**Fig. 2:**
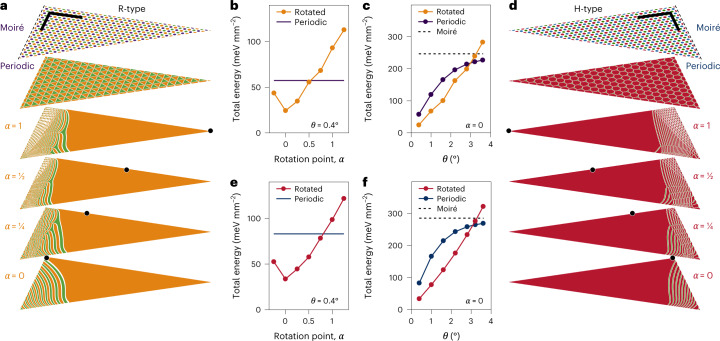
Mesoscopic reconstruction in finite size simulations. **a**,**d**, Maps of reconstructed domains in triangular tips of R-type (**a**) and H-type (**d**) heterostacks with a twist angle *θ* = 0.4° (only triangle halves are shown in the projections; the scale bars are 200 nm). The top maps show moiré patterns without reconstruction (delimited by dashed lines from the moiré core of the HBL), whereas the maps below show periodic reconstruction and mesoscopically reconstructed domain patterns obtained for different zero-twist deformations around the points marked by black dots at dimensionless positions *α* (note that the orange ${R}_{h}^{X}$ and green ${R}_{h}^{M}$ domains can interconvert due to similar adhesion energies^35^). **b**,**e**, Total areal energy for R (**b**) and H (**e**) at *θ* = 0.4°, and different untwisting points *α* (the energy of the respective periodic patterns is shown by solid lines). **c**,**f**, Total areal energy of periodic and optimally reconstructed (for *α* = 0) patterns for different twist angles *θ* in R (**c**) and H (**f**) (the energy of the respective moiré patterns is indicated by the dashed lines). Source data

The results of our numerical simulations are shown for R- and H-type HBLs with a twist of *θ* = 0.4° (Fig. 2a,d). The top two maps illustrate the ideal moiré and periodically reconstructed patterns, whereas the four maps below show reconstruction patterns after optimization of initial displacement fields that untwist the HBL around a rotation centre indicated by black points and labelled by a dimensionless coordinate *α* = 1, 0.5, 0.25, 0. In all cases, optimization yields mesoscopic reconstruction into 2D domains of energetically favoured stackings at the triangle tip (${R}_{h}^{X}$ or ${R}_{h}^{M}$ and ${H}_{h}^{h}$ in R- and H-stacks). These extended domains are flanked by 1D stripes, which merge into 0D domain arrays.

As expected, optimality reconstructed patterns minimize the total energy of the system according to our simulations shown for R-type (Fig. 2b,c) and H-type (Fig. 2e,f) HBLs. For *θ* = 0.4°, the total energy (normalized by the triangle area) in both stackings is reduced by a factor of 10 and 2 below the energies of ideal moiré and periodic limits, respectively (solid lines in Fig. 2b,e), on reconstruction after optimal rotation at *α* = 0. The global energy minimum—obtained for the rotation point at the borderline between the moiré core and the triangle base—features the most direct transition from the 2D domain through 1D stripes to the 0D core. As the rotation point is moved towards the tip (via 0 < *α* < 1), 0D regions emerge at the border and the energy gain decreases, passing the threshold of periodic reconstruction at *α* ≃ 0.5 (0.75) in the R (H) heterostack but remaining well below the moiré lattice energy throughout. Remarkably, for the optimally relaxed tip around *α* = 0, mesoscopic reconstruction remains energetically favourable for twist angles up to *θ* = 3° for both stackings (Fig. 2c,f), in accordance with conclusions from recent Raman spectroscopy studies^46^.

### Excitons in reconstructed R-heterostacks

**Fig. 3:**
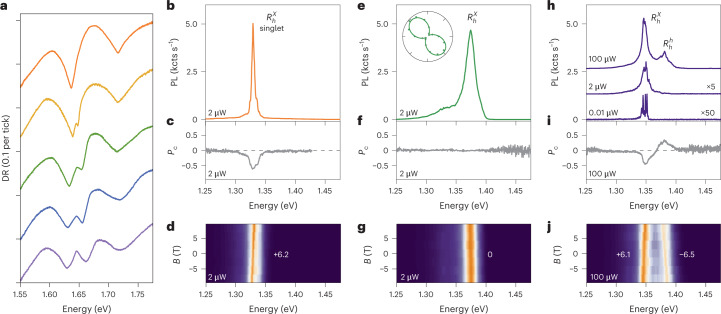
Spectral characteristics of excitons in reconstructed R-type MoSe_2_–WSe_2_ HBLs. **a**, Evolution of differential reflection (DR) spectra of intralayer excitons following gradual displacement from a bright to dark region (shown from top to bottom for positions indicated by the black dots in Supplementary Fig. 9a). The peak multiplicity is a hallmark of nanoscale reconstructed domains. **b**–**d**, Interlayer exciton PL (**b**), *P*_c_ (**c**) and dispersion in a perpendicular magnetic field *B* (**d**), characteristic of extended 2D domains. The magneto-luminescence data were recorded under linearly polarized excitation with *σ*^+^ detection to determine the *g*-factor values from linear slopes. **e**–**g**, Same as **b**–**d** but for regions of 1D stripes with a large degree of linear polarization (as shown in the inset). **h**–**j**, Same as **b**–**d** but in a dark sample region of 0D domains (PL spectra shown with offsets and different scaling for excitation powers of 100, 2 and 0.01 μW). All data were recorded on sample 2 (Supplementary Fig. 9) ; *g*-factor values with least-square error bars were obtained from linear fits to the data shown in Supplementary Fig. 16. Source data

With the intuition for mesoscopic reconstruction, we revisit distinct spectroscopic features of R-stacks in the intralayer and interlayer exciton spectral range with differential reflection and PL, respectively. The differential reflection spectra—proportional to intralayer exciton absorption—evolve as the observation spot is moved from bright to dark PL areas (Fig. 3a). Following displacement, the differential reflection resonances around the MoSe_2_ and WSe_2_ intralayer exciton transitions at 1.64 and 1.71 eV on the bright spots (top spectrum) gradually develop split resonances and broadening that is most pronounced in the darkest areas (bottom spectrum). This evolution reflects the presence of only one registry in bright spots, and modulation of the intralayer exciton energy by alternating stackings in the darkest spots. As resonant hybridization in MoSe_2_–WSe_2_ heterostacks is strongly inhibited by large band offsets, multi-peak differential reflection spectra result from different intralayer exciton energies within the different registries probed by the optical spot.

Bearing in mind the absence of peak multiplicity in the differential reflection spectra of bright spots, the corresponding characteristics of interlayer exciton PL (Fig. 3b–d) are readily explained. Due to locally extended reconstruction, only lowest-energy ${R}_{h}^{X}$ singlet excitons contribute to PL (Fig. 3b), with a peak at 1.33 eV and full-width at half-maximum linewidth of 6 meV, a negative *P*_c_ (Fig. 3c) and a positive *g*-factor of ~6 (Fig. 3d), as in aligned HBLs^21^. Such characteristics are frequently observed at sample edges and tips (as in Fig. 1c) where large-scale reconstruction is energetically most favourable (as in the reconstructed maps of Fig. 2a).

The PL from spatially neighbouring regions is indicative of quantum wire domains^44^, with blue-shifted emission at around 1.36 eV, a high *P*_l_ (inset in Fig. 3e), and a vanishing *P*_c_ and *g*-factor value (Fig. 3f,g). In R-stacks, quantum wires are formed by alternating optically bright ${R}_{h}^{X}$ and dark ${R}_{h}^{M}$ domains. The related 1D confinement of interlayer excitons in stripes of lower-energy ${R}_{h}^{X}$ domains flanked by potential walls of higher-energy ${R}_{h}^{M}$ states not only breaks the threefold rotational symmetry of the exciton wave function (thereby admixing *K* and ${K}^{{\prime} }$ valleys, and obliterating both *P*_c_ and *g*-factors in perpendicular magnetic field), it is also responsible for the blue-shift in PL energy. Spot-to-spot variations between sample regions of high *P*_l_, with varying orientations of linear polarization axes, are consistent with the diversity of stripe geometries. A prominent example is the left corner of the R-type triangle in Fig. 1c, in which the bright spot of a large ${R}_{h}^{X}$ domain with *P*_c_ ≃ −1 is encompassed by quantum wire regions with *P*_l_ ≃ ±1.

Quantum confinement is also prominent in R-type regions of 0D arrays that have much reduced PL intensity and spectrally narrow lines at low excitation powers (note the scaling by 5 and 50 for the spectra at 2 and 0.01 μW, respectively, in Fig. 3h), with characteristic negative *P*_c_ (Fig. 3i) and *g*-factors of around ±6 (Supplementary Figure 14b)^11^. Depending on the actual spot, such quantum dot lines with a full-width at half-maximum well below 1 meV can be observed within spectrally narrow or broad windows of 10 or 100 meV (as seen in Fig. 3h and Fig. 1e, respectively) above the peak of extended ${R}_{h}^{X}$ domains at 1.33 eV. Obviously, regions of 0D arrays increase the energy of interlayer excitons by quantum confinement in nanoscale domains of ${R}_{h}^{X}$ stacking, with potential barriers formed by adjacent ${R}_{h}^{M}$ domains, whereas the variations in PL energies relate to different strengths of confinement in quantum boxes of varying size.

The spectrally dispersed PL from inhomogenously reconstructed arrays merges at elevated excitation powers (spectra at 2 μW in Figs. 3h and 1e) into structured PL peaks of sub-ensembles grouped by similar length scales. Reconstructed 0D arrays with lateral homogeneity give rise to a narrow ensemble of emission energies (within 10 meV as in Fig. 3h), which allows us to observe hot luminescence with a positive *P*_c_ and negative *g*-factor of −6.5 (Fig. 3i,j at 100 μW excitation power). These features—absent in the spectra of 1D and 2D domains—correspond to ${R}_{h}^{h}$ singlet interlayer excitons ~50 meV above ${R}_{h}^{X}$ states. Such a prominent contribution of ${R}_{h}^{h}$ stacking to the PL is surprising, as theory predicts vanishingly small areas for this non-optimal stacking^34–36^. The sizable PL with ${R}_{h}^{h}$ characteristics therefore either implies areas of ${R}_{h}^{h}$ domains that are larger than anticipated from theory, or that their exciton population is favoured by population-feeding pathways from excited states or nearby ${R}_{h}^{X}$ domains.

### Excitons in reconstructed H-type heterostacks

**Fig. 4:**
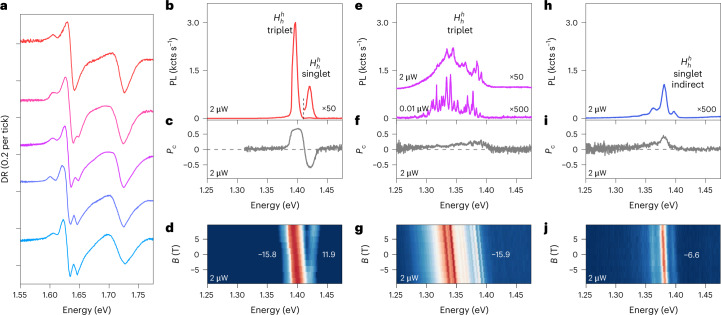
Spectral characteristics of excitons in reconstructed H-type MoSe_2_–WSe_2_ HBL. **a**, Evolution of differential reflection spectra of intralayer excitons following displacement from a bright to dark region (shown from top to bottom curves for positions indicated by red dots in Supplementary Fig. 10a) with peak multiplicity stemming from reconstructed nanoscale domains. **b**–**j**, Interlayer exciton PL (**b, e, h**), *P*_c_ (**c, f, i**) and *B*-field dispersion (**d, g, j**) for three representative positions. The magneto-luminescence data were recorded under linearly polarized excitation with *σ*^+^ detection to determine the *g*-factor values from linear slopes. Spots with bright PL (as in **b**) feature triplet and singlet peaks with opposite *P*_c_ signs and characteristic *g*-factors of about −16 and 12. Sample positions with low PL intensity exhibit structured spectra (as in **e** and **h**) with reduced *P*_c_. Their characteristics differ both in spectral profiles and *g*-factors. Data in **e**–**g** are from sample 2, whereas all other data are from sample 3 (see Supplementary Fig. 10). The *g*-factor values with least-square error bars were obtained from linear fits to the data in Supplementary Fig. 17. Source data

Despite similarities, some aspects of mesoscopic reconstruction in H-type HBLs are distinct. The differential reflection spectra of intralayer excitons in the bright and dark PL regions shown in Fig. 4a (the feature at 1.61 eV is due to residual doping in this sample) lead to the same conclusion as for the R-type spectra of Fig. 3a, that is, multi-peak intralayer differential reflection resonances in regions of dark PL are absent in the regions of bright PL. For the latter, the atomic registry of reconstructed 2D domains is ${H}_{h}^{h}$—unrivalled by other registries in terms of energy-optimizing stacking^31,34,35^. The respective PL spectrum of interlayer excitons (Fig. 4b) is therefore simple, featuring only the emission peaks of triplet and singlet configurations at 1.40 and 1.42 eV with positive and negative *P*_c_ (Fig. 4c), and *g*-factors of −15.8 and 11.9 (Fig. 4d), respectively^21,47,48^.

As for R-type HBLs, the differential reflection spectra exhibit an increasingly pronounced splitting of the MoSe_2_ intralayer exciton resonance in dark regions of H-type samples (bottom spectra in Fig. 4a). In the respective PL spectra (Fig. 4e), for low and moderate excitation powers (0.01 and 2 μW, respectively) we again observe spectrally sharp peaks developing into a multi-peak ensemble of 0D arrays with an inhomogeneous size distribution of nanoscale domains in optimal ${H}_{h}^{h}$ stacking. The positive *P*_c_ (Fig. 4f) and the *g*-factor values (Fig. 4g) confirm ${H}_{h}^{h}$ stacking as the origin of the emission.

For this stacking, theoretical estimates (Table 1) place its optically bright interlayer exciton states at the top of the energy hierarchy above the dark ${H}_{h}^{M}$and ${H}_{h}^{X}$states. This energetic ordering is responsible for the absence of 1D features found in R-stacks: excitons in reconstructed ${H}_{h}^{h}$ domains are not bound by potential barriers that would mix *K* and ${K}^{{\prime} }$ states as in 1D quantum wires of R-stacks (note the absence of areas with high *P*_l_ throughout the map of H-type triangle in Fig. 1c). Our theory predicts that reconstructed ${H}_{h}^{h}$ domains preserve luminescent exciton population on spatially extended plateaus, whereas the PL intensity decreases in regions of 0D arrays (note the scaling factors of 50 and 500 in Fig. 4e) due to population drain into lower-energy states of the surrounding ${H}_{h}^{M}$and ${H}_{h}^{X}$domains with much reduced optical activity and domain areas. Consistently, nanoscale domain formation is accompanied by PL red-shifts of up to 100 meV below the ${H}_{h}^{h}$ triplet peak at 1.4 eV.

For completeness, in Fig. 4h–j we show the PL characteristics of a dark area in an H-stack with about 3° twist. The heterostack is not entirely prone to reconstruction, with tips and edges exhibiting signatures of bright ${H}_{h}^{h}$ domains (see Supplementary Fig. 10); however, the dark areas of the sample exhibit very different features than reconstructed 0D arrays discussed above, implying that the HBL largely maintains its canonical moiré structure. As shown in Fig. 4h, the PL at 2 μW excitation power is reduced by another factor of 10 (note the scaling factor of 500), with a positive *P*_c_ (Fig. 4i) and a negative *g*-factor of −6.6 (Fig. 4j) that has no counterpart in the realm of zero-momentum ${K}^{{\prime} }K$ interlayer excitons of H-type registries yet is characteristic of finite-momentum *KK* exciton states (see Supplementary Table 2). The peaks with equidistant energy spacing of 16 meV up to the sixth order (Supplementary Fig. 18) are indicative of interlayer exciton–polaron formation^49^. In this regime, the heterostructure with layer- and valley-separated excitons dressed by strong exciton–phonon coupling is rendered momentum-dark, with luminescent population decay of exciton–polaron states mediated by a series of phonon replicas.

## Conclusion

Our insight into mesoscopic reconstruction in MoSe_2_–WSe_2_ HBLs identifies co-existing domains of different dimensionality and exciton characteristics, with direct evidence provided by one-to-one correlations between local spectral features and sample morphology (see Supplementary Note 5). Extended HBLs with small twist exhibit luminescent singlet and triplet interlayer excitons in extended 2D domains of energetically favourable ${R}_{h}^{X}$and ${H}_{h}^{h}$ registries which occur mainly near sample edges or line defects. Stripes of 1D domains connect such 2D areas to arrays of nanometre-sized 0D domains with split intralayer exciton resonances (see Supplementary Note 6) and spectrally narrow lines of interlayer excitons. In contrast, HBLs locked in moiré patterns host optically dark exciton–polarons. The broad evidence for mesoscopic reconstruction in bilayer graphene^30,50,51^, hBN^52,53^ and CrI_3_ ferromagnets^54,55^ suggests that the phenomenon is universal across samples and devices of layered van der Waals stacks. Ultimately, the understanding and control of mesoscale reconstruction will enable future realizations of layered quantum materials with tailored electronic, optical and magnetic properties.

## Methods

### Sample fabrication

Monolayers of MoSe_2_ and WSe_2_ were either mechanically exfoliated from bulk crystals (HQ Graphene) or obtained from CVD synthesis. Thin flakes of hBN were exfoliated from bulk crystals (National Institute for Materials Sscience). Fully hBN-encapsulated MoSe_2_–WSe_2_ heterobilayers were prepared with a polycarbonate/polydimethylsiloxane stamp by dry-transfer^56^. First, a layer of hBN was picked up, followed by the MoSe_2_ and WSe_2_ monolayers, and then a capping layer of hBN. The pick-up temperatures for the hBN flakes, and the WSe_2_ and MoSe_2_ monolayers was around 50 °C, 130 °C and 100 °C, respectively. The monolayers were aligned to 0° (R-type) or 60° (H-type) by selecting adjacent straight edges with angles of either 60° or 120°. The precision of alignment was limited to below 1°. The heterostacks were finally released onto 300 nm SiO_2_/Si substrates at a temperature of 180 °C. To avoid thermally activated rotation of TMD layers, no thermal annealing was performed unless specified otherwise.

For direct comparison of R- and H-stackings, two MoSe_2_–WSe_2_ samples—with both alignment configurations—were fabricated. One sample was stacked from CVD-grown triangular single-layer crystals, with two MoSe_2_ monolayers of opposite orientation placed onto a large WSe_2_ monolayer to form two HBL regions of R- and H-type. It has been shown that zigzag edges are predominant in CVD-grown triangular TMD monolayers^57^. Therefore, in the sample shown in Fig. 1b, the regions with parallel and antiparallel edges of MoSe_2_ monolayer triangles, to the edge of the large WSe_2_ triangle are R- and H-type, respectively. Other samples were obtained from mechanical exfoliation-stacking by the tear-and-stack method^58^, using a large WSe_2_ monolayer to pick up a part of a MoSe_2_ monolayer, and the remaining part following a rotation by 60°.

### Optical spectroscopy

Cryogenic PL and differential reflection measurements were conducted using a home-built confocal microscope in back-scattering geometry. The samples were loaded into a closed-cycle cryostat (attocube systems, attoDRY1000) with a base temperature of 3.2 K. The cryostat was equipped with a superconducting magnet providing magnetic fields of up to 9 T in a Faraday configuration. Piezo-stepping and scanning units (attocube systems, ANPxyz and ANSxy100) were used for sample positioning with respect to a low-temperature apochromatic objective. For PL measurements, a titanium:sapphire laser (Coherent, Mira) in continuous-wave mode was employed to excite the samples and was tuned to the resonance of intralayer exciton transition in WSe_2_ monolayer at 725 nm. For differential reflection measurements, a stabilized tungsten–halogen lamp (Thorlabs, SLS201L) was used as a broadband light source. The PL or reflection signal were spectrally dispersed by a monochromator (Roper Scientific, Acton SP2500 or Acton SpectraPro 300i with a 300 grooves per millimetre grating) and detected by a liquid nitrogen cooled or Peltier cooled charge-coupled device (Roper Scientific, Spec-10:100BR or Andor, iDus 416). A set of linear polarizers (Thorlabs, LPVIS), half- and quarter-waveplates (B. Halle, 310−1,100 nm achromatic) mounted on piezo-rotators (attocube systems, ANR240) were used to control the polarization in excitation and detection. The differential reflection spectra were obtained by normalizing the reflected spectra from the HBL region (*R*) to that from the sample region without MoSe_2_ and WSe_2_ layers (*R*_0_) as DR = (*R* − *R*_0_)/*R*_0_. Time-resolved PL was excited with a wavelength-tunable supercontinuum laser (NKT Photonics, SuperK Extreme and SuperK Varia) at 725 nm with a pulse duration of 6 ps and repetition rates down to 0.625 MHz, detected with a silicon avalanche photodiode (PerkinElmer, SPCM-AQRH-15) and correlated with a time-correlating single-photon counting module (PicoQuant, PicoHarp 300).

### SEM imaging

Scanning electron microscopy imaging of reconstruction in MoSe_2_–WSe_2_ HBLs was performed with a Raith eLine system. Further technical details are provided in Supplementary Note 1.

### Theoretical modelling

Mesoscopic reconstruction was modelled in numerical simulations by discretizing the displacement field of the HBL lattice with a square mesh, followed by optimization of the total energy with the trust-region algorithm implemented in MATLAB^59^. Density functional theory calculations of high-symmetry HBL stackings with relaxed lattices were performed with the Vienna ab-initio simulation package with the PBEsol exchange-correlation functional. Technical details are provided in Supplementary Notes 2 and 7.

## Online content

Any methods, additional references, Nature Portfolio reporting summaries, source data, extended data, supplementary information, acknowledgements, peer review information; details of author contributions and competing interests; and statements of data and code availability are available at 10.1038/s41565-023-01356-9.

## Supplementary information

Supplementary Information Supplementary Notes 1–7, Figs. 1–29 and Tables 1–3.

## Data Availability

Additional data that support the findings of this study are available from the corresponding authors on reasonable request. Source Data are provided with this paper.

## Source data

Source Data Fig. 1 Statistical Source Data.
Source Data Fig. 2 Statistical Source Data.
Source Data Fig. 3 Statistical Source Data.
Source Data Fig. 4 Statistical Source Data.

## Code availability

The codes that support the findings of this study are available from the corresponding authors on reasonable request.
