# Supplementary material for: Excitons in mesoscopically reconstructed moiré heterostructures
Source: Nat Nanotechnol. 2023 Mar 27;18(6):572–9. doi: 10.1038/s41565-023-01356-9 (PMC10275756; doi:10.1038/s41565-023-01356-9)
Supplement: Supplementary file 1 — Supplementary Notes 1–7, Figs. 1–29 and Tables 1–3. [file 41565_2023_1356_MOESM1_ESM.pdf]

nature nanotechnology

Article

https://doi.org/10.1038/s41565-023-01356-9

Excitons in mesoscopically reconstructed moiré heterostructures

In the format provided by the
authors and unedited

CONTENTS

Note **1**: Mesoscopic reconstruction in SEM 2

Note **2**: Modelling of mesoscopic reconstruction 4

Note **3**: Sample characteristics and spectral features 10

Note **4**: Time-resolved photoluminescence of reconstructed domains 24

Note **5**: Direct correlation between domain patterns and optical properties 28

Note **6**: Analysis of intralayer exciton absorption spectra in reconstructed domains 35

Note **7**: Density functional theory calculations 38

Supplementary References 41

Supplementary Note 1: Mesoscopic reconstruction in SEM

To image mesoscopic lattice reconstruction in $MoSe_2$-WSe_2 HBLs (as in Fig. 1f,g of the main text) we used the secondary electron (SE) imaging technique in low-energy scanning electron microscopy (SEM) initially pioneered on SiC crystals [1] and recently adopted to twisted WSe_2 homobilayers [2]. In brief, the technique is based on out-lens detection of SEs emitted from atoms near the crystal surface by inelastic scattering of incoming primary electrons. In a TMD bilayer, the top and bottom hexagonal lattices form an effective cavity for electrons, rendering the scattering probability for the incoming beam in the cavity and thus the generation of SE dependent on the relative atomic positions in given stacking configurations. In optimized experimental geometries, the contrast of SE yield under channeling conditions allows to discriminate domains of different stacking configurations [2]. We adopted the technique to image R- and H-type $MoSe_2$-WSe_2 HBLs. The samples were prepared by the same method as for optical spectroscopy yet without hBN encapsulation. For both R- and H-type HBLs, the measurements were performed in a Raith eLine system (equipped with an Everhart-Thornley detector)at 1 keV electron beam energy and normal incidence for H-type and $\sim 38°$ tilt for R-type samples. Respective SEM images of H- and R-type $MoSe_2$-WSe_2 HBLs are shown in Supplementary Fig. 1 and 2.

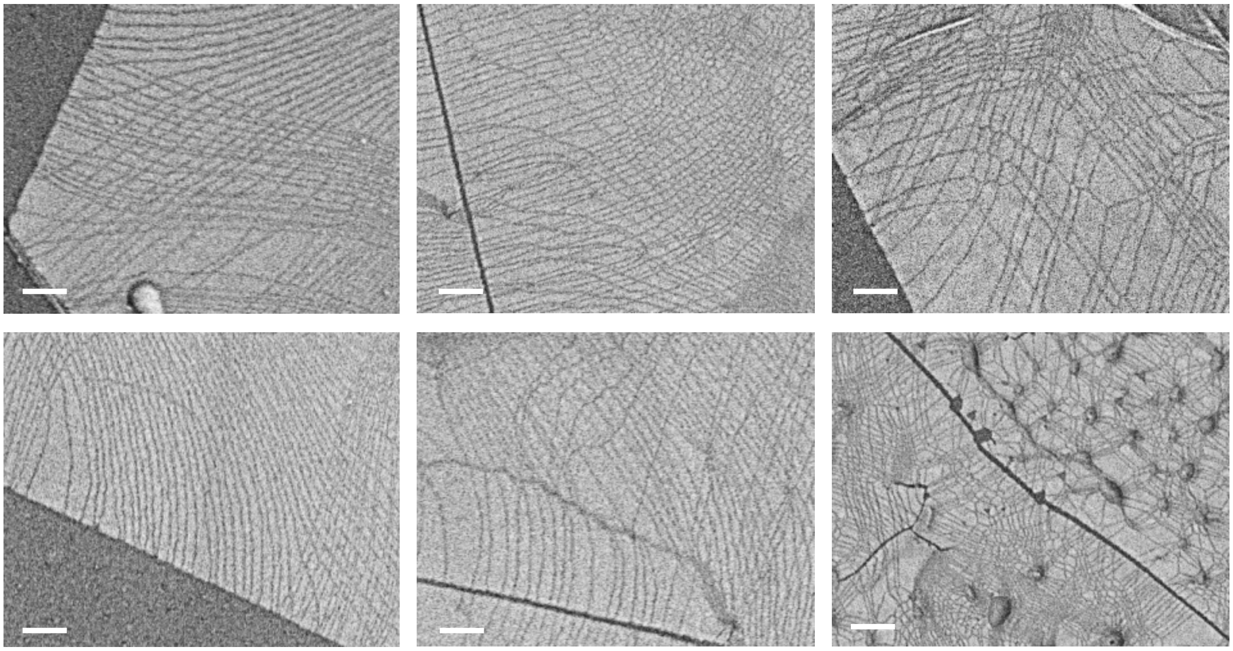

Supplementary Figure 1. SEM images of nearly aligned H-type $MoSe_2$-WSe_2 HBLs. The scale bars are 600 nm.

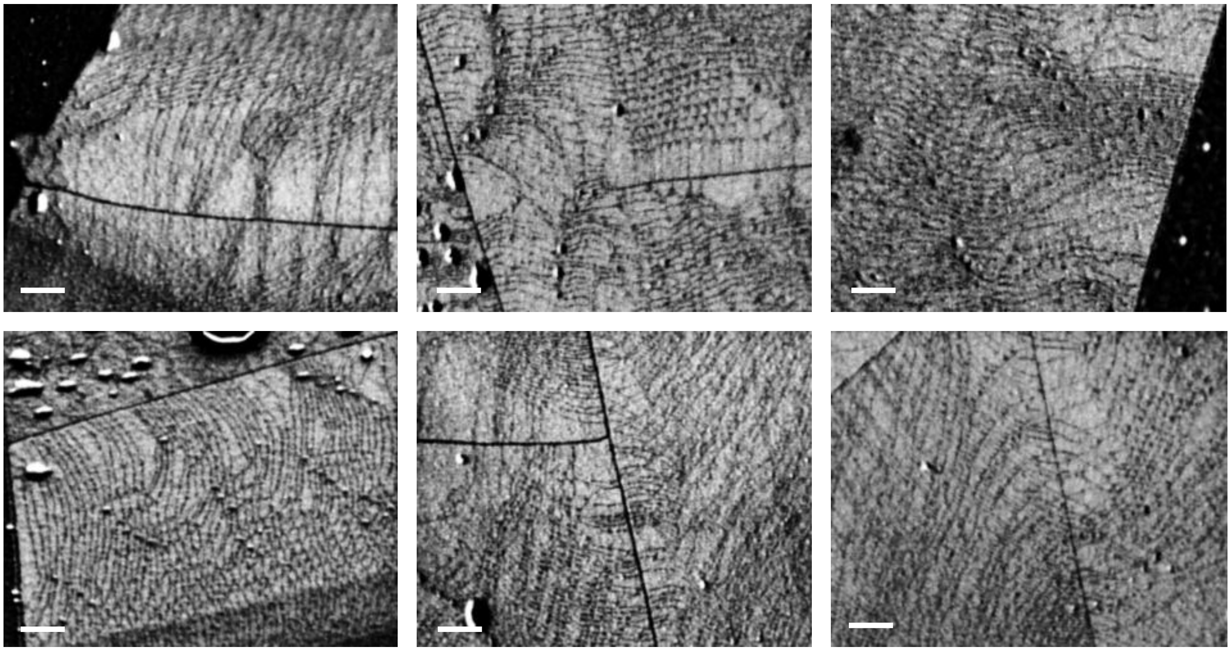

Supplementary Figure 2. SEM images of nearly aligned R-type $MoSe_2$-WSe_2 HBLs. The scale bars are 600 nm.

The images illustrate for nearly-aligned H- and R-type $MoSe_2$-WSe_2 HBLs clear departures from canonical moiré patterns or uniformly reconstructed periodic domains. They actually exhibit large variations in domain shape and size with the following common features: from the tips and edges to the cores, the reconstructed pattern evolves from large micron-sized two-dimensional (2D) domains to elongated one-dimensional (1D) stripes which merge into a network of zero-dimensional (0D) arrays with dimensions well below 100 nm with variations in domain shape and size. In addition to line defects such as tips and edges, local strain such as interfacial bubbles in the bottom right panel of Fig. 1 can also give rise to mesoscopic reconstruction. The main difference between H- and R-type HBLs is that only one staking (H_h^h) is optimal in the former, whereas two stackings (R_h^X and R_h^M) yield optimally reconstructed domains in the latter.

Supplementary Note 2: Modelling of mesoscopic reconstruction

Reconstruction of $MoSe_2$-WSe_2 HBL is driven by the interplay of interlayer adhesion energy and strain. The sum of the intralayer and interlayer energies of the lattice is given by the integral over the HBL area S as [3]:

$$\mathcal{E} = \int_S \left[U(\mathbf{r}) + W_s(\mathbf{r})\right] \mathrm{d}\mathbf{r}, \tag{1}$$

where $U(\mathbf{r})$ and $W_s(\mathbf{r})$ are the respective intralayer and interlayer strain energy densities which depend on the in-plane displacements in $MoSe_2$ and WSe_2 layers. Assuming equal elastic properties and lattice constants in both layers [4], the displacement fields are given by:

$$\mathbf{u}_{\mathrm{WSe}_2}(\mathbf{r}) = -\mathbf{u}_{\mathrm{MoSe}_2}(\mathbf{r}) = \frac{\mathbf{u}(\mathbf{r})}{2} \equiv \left(\frac{u_x(\mathbf{r})}{2}, \frac{u_y(\mathbf{r})}{2}\right). \tag{2}$$

The intralayer strain energy density is given by:

$$U(\mathbf{r}) = \frac{1}{2}\left[\frac{\lambda}{2}\left(\sum_i u_{ii}(\mathbf{r})\right)^2 + \mu \sum_{ij} u_{ij}^2(\mathbf{r})\right] \tag{3}$$

with the strain tensor:

$$u_{ij}(\mathbf{r}) = \frac{1}{2}[\partial_j u_i(\mathbf{r}) + \partial_i u_j(\mathbf{r})], \tag{4}$$

where the first and second Lamé parameters λ and μ are obtained by averaging over the respective parameters in $MoSe_2$ and WSe_2 MLs.

The interlayer term $W_s(\mathbf{r}) = V_s(\mathbf{r}) - \varepsilon Z_s^2(\mathbf{r})$ quantifies the adhesion energy density defined via the expressions [5]:

$$V_s(\mathbf{r}) = \sum_{n=1}^{3}\{A_1 e^{-Qd_0}\cos\phi_n(\mathbf{r}) + A_2 e^{-Gd_0}\sin\left[\phi_n(\mathbf{r}) + \varphi_s\right]\}, \tag{5}$$

$$Z_s(\mathbf{r}) = \frac{1}{2\varepsilon}\sum_{n=1}^{3}\{QA_1 e^{-Qd_0}\cos\phi_n(\mathbf{r}) + GA_2 e^{-Gd_0}\sin\left[\phi_n(\mathbf{r}) + \varphi_s\right]\}, \tag{6}$$

where the index $s = R, H$ denotes R-type and H-type stackings with the twist angle between the top and bottom layers θ, and $\phi_n(\mathbf{r}) = \mathbf{g}_n \cdot \mathbf{r} + \mathbf{G}_n \cdot \mathbf{u}(\mathbf{r})$ is defined through $\mathbf{g}_n = -\theta\, \mathbf{e}_z \times \mathbf{G}_n$ with the vectors of the first star of the reciprocal lattice $\pm\mathbf{G}_n$ $(n = 1, 2, 3)$ and $G = |\mathbf{G}_n| = 4\pi/(\sqrt{3}a)$ with the lattice constant a, $\varphi_{\mathrm{R}} = \pi/2$, $\varphi_{\mathrm{H}} = 0$, and $Q = \sqrt{G^2 + \rho^{-2}}$; ε, A_1, A_2, d_0, and ρ are fitting parameters. We use all material and fitting parameters for $MoSe_2$-WSe_2 HBL from Ref. [3]: $a = 0.329$ nm, $d_0 = 0.69$ nm, $\rho = 0.052$ nm, $\lambda = 222$ eV/nm^2, $\mu = 306$ eV/nm^2, $A_1 = 77621500$ eV/nm^2, $A_2 = 84739$ eV/nm^2, and $\varepsilon = 189$ eV/nm^4.

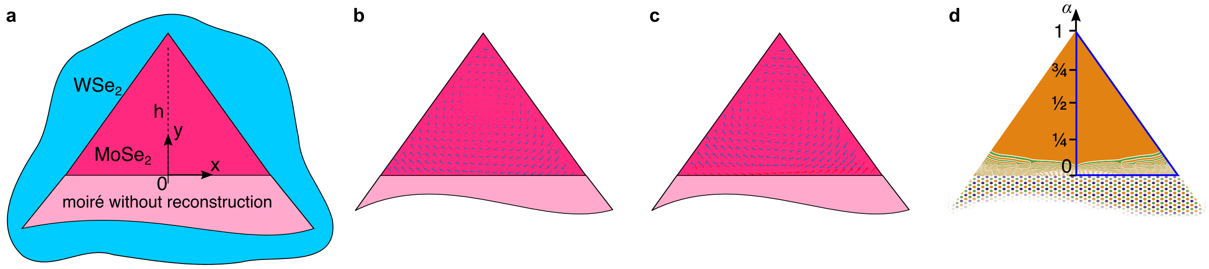

Supplementary Figure 3. **a**, Geometry of triangular $MoSe_2$-WSe_2 HBL tip used in simulations of mesoscopic reconstruction, with pink and blue colors denoting the top $MoSe_2$ ML and the bottom WSe_2 ML, respectively. **b** and **c**, Illustration of the initial and final displacement fields $\mathbf{u}^{\mathrm{i}}$ and $\mathbf{u}^{\mathrm{f}}$, respectively, for the reconstruction pattern of R-type HBL with $\alpha = 0.5$ shown in **d**.

In the next step, we assume an equilateral triangle for the shape of the HBL area S. Supplementary Fig. 3a shows the triangle baseline placed on the x-axis as a borderline to the moiré region in the HBL with twist angle θ and without reconstruction. At the borderline, the displacement field is zero, $u_x(x,0) = u_y(x,0) = 0$, whereas the remaining two sides of the triangle are free from boundary conditions. We assume that the y-axis bisects the angle θ and divides the equilateral triangle into two right-angled triangles, yielding two constrains:

$$u_x(x,y) = u_x(-x,y), \tag{7}$$

$$u_y(x,y) = -u_y(-x,y). \tag{8}$$

Finally, we discretize the displacement field $\mathbf{u}(\mathbf{r})$ with a square mesh, replace the integration in Eq. (1) by the summation over the triangle area, and express the derivatives in Eq. (4) through the finite differences method. The density of the square mesh (we choose 288 moiré unit cells in all simulations of equilateral triangles) determines the number of discretization parameters (*i.e.* if the mesh divides the height of triangle h into N equal sectors, the displacement field is defined by $\sim (N+1)^2/\sqrt{3}$ parameters). To find the parameter set that minimizes the total energy in Eq. (1), we use the trust-region algorithm implemented in the Optimization Toolbox™ of MATLAB® to determine the local energy minimum of the final displacement field $\mathbf{u}^{\mathrm{f}}$ as a function of the initial displacement field $\mathbf{u}^{\mathrm{i}}$ set prior to reconstruction. In all calculations, we monitor the convergence of numerical results.

In Fig. 2a,d of the main text we used the following initial sets of displacement fields. The ideal moiré case was obtained with the initial set $\mathbf{u}^{\mathrm{i}} = \mathbf{0}$ (twisted HBL with $\theta = 0.4°$) and without reconstruction. The periodic case illustrates the final set after the reconstruction

with the initial field $\mathbf{u}^{\mathrm{i}} = \mathbf{0}$. All remaining cases show the final sets with the rotated initial displacement:

$$u_x^{\mathrm{i}} = \theta(y - \alpha h), \tag{9}$$

$$u_y^{\mathrm{i}} = -\theta x, \tag{10}$$

which realizes the rotation of layers by $\pm\theta/2$ around the point $(0, \alpha h)$ and leads to untwisted HBL regions when the stacking expands from the rotation point to the entire area of the triangle. To denote these initial displacement fields we use the dimensionless y-coordinate of the rotation point, α.

Supplementary Fig. 3 illustrates the reconstruction in the tip of a triangular R-type HBL (Supplementary Fig. 3a) for an initial field displacement $\mathbf{u}^{\mathrm{i}}$ with a rotation point at $\alpha = 0.5$ (Supplementary Fig. 3b). The optimally reconstructed tip is described by the final displacement field $\mathbf{u}^{\mathrm{f}}$ (Supplementary Fig. 3c) yielding the characteristic mesoscopic domain network (Supplementary Fig. 3d). To complement the data of Fig. 2a,d of the main text, we show in Supplementary Figs. 4 and 5 the vector fields of the initial and final displacements, $\mathbf{u}^{\mathrm{i}}$ and $\mathbf{u}^{\mathrm{f}}$, and by red arrows their difference $\mathbf{u}^{\mathrm{i}} - \mathbf{u}^{\mathrm{f}}$ after reconstruction. Additionally, we show the corresponding maps of intralayer and interlayer energies.

To illustrate the generalization of our results for more than one rotation point we show in Supplementary Fig. 6 reconstruction maps for R- and H-type HBLs with a twist angle of 3°. The top panel shows periodic reconstruction, and the central panel illustrates reconstruction with one rotated point at $\alpha = 0$. The maps in the bottom panels were calculated for two rotation points at $\alpha = 0$ and $\alpha = 1/12$ with the initial displacement field:

$$\mathbf{u}^{\mathbf{i}} = \begin{cases} \alpha = 0, & \text{for } 0 \leq y \leq 7h/12; \\ \mathbf{0}, & \text{for } 7h/12 < y \leq 2h/3; \\ \alpha = 1/12, & \text{for } 3h/4 < y \leq h. \end{cases} \tag{11}$$

This initial displacement field realizes three areas upon reconstruction: two untwisted domains around points with $\alpha = 0$ and $\alpha = 1/12$ with zero initial displacement in between. The resulting patterns exhibit regions of both periodically reconstructed and rotated patterns. This procedure can be expanded to model realistic samples with complex reconstruction patterns as in Supplementary Fig. 1 and 2 by taking into account multiple rotation points for a given sample geometry and strain distribution.

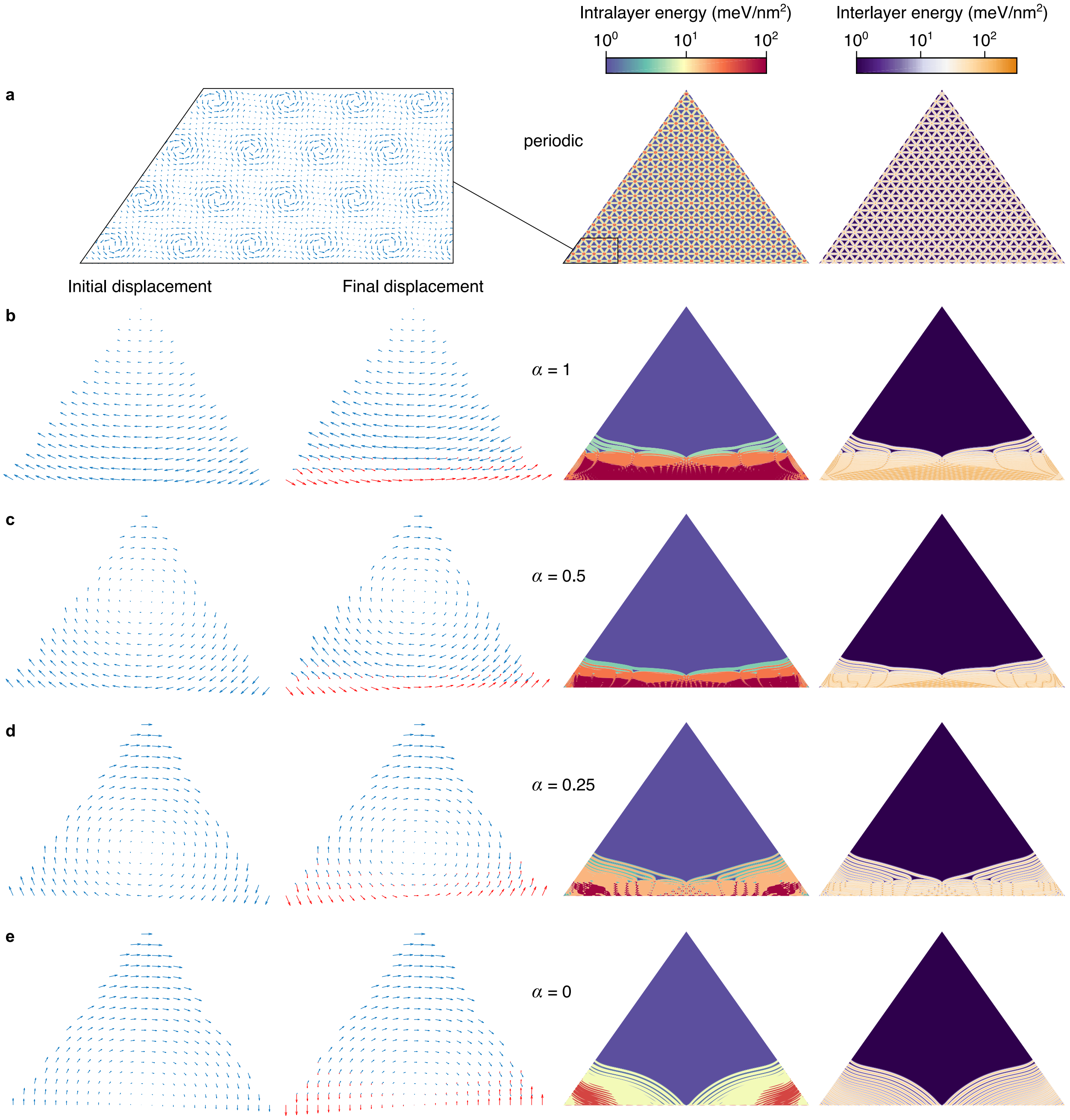

Supplementary Figure 4. $\mathbf{a}-\mathbf{e}$, Initial and final displacement fields as well as intra- and interlayer energies of reconstructed patterns for R-type HBLs in Fig. 2a of the main text. For the periodic case, **a**, we show only the final displacement field. The panels $\mathbf{b}-\mathbf{e}$ relate to the initial displacement fields with rotation point at $\alpha = 1, 0.5, 0.25, 0$.

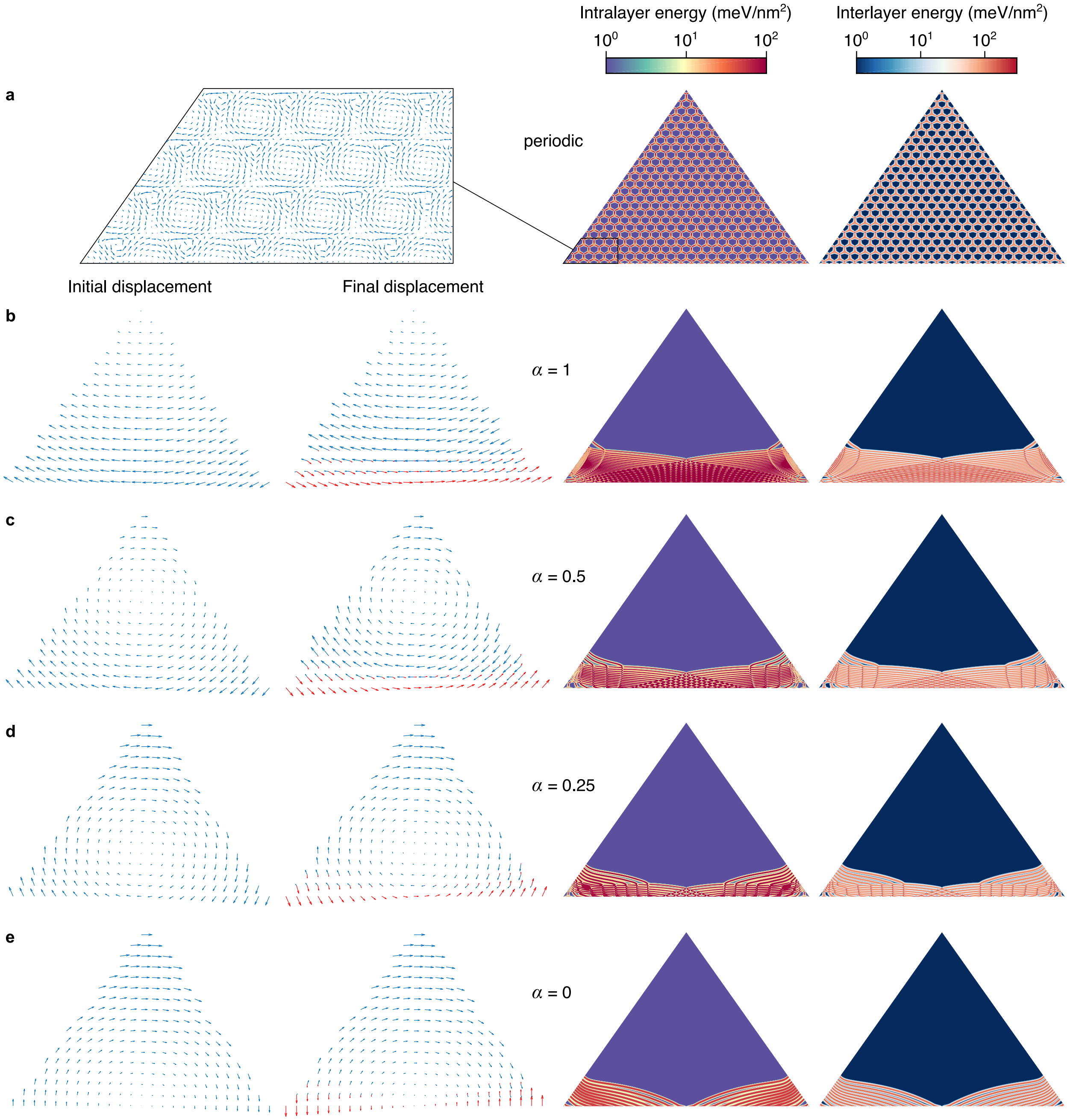

Supplementary Figure 5. $\mathbf{a}-\mathbf{e}$, Initial and final displacement fields as well as intra- and interlayer energies of reconstructed patterns for H-type HBLs in Fig. 2d of the main text. For the periodic case, **a**, we show only the final displacement field. The panels $\mathbf{b}-\mathbf{e}$ relate to the initial displacement fields with rotation point at $\alpha = 1, 0.5, 0.25, 0$.

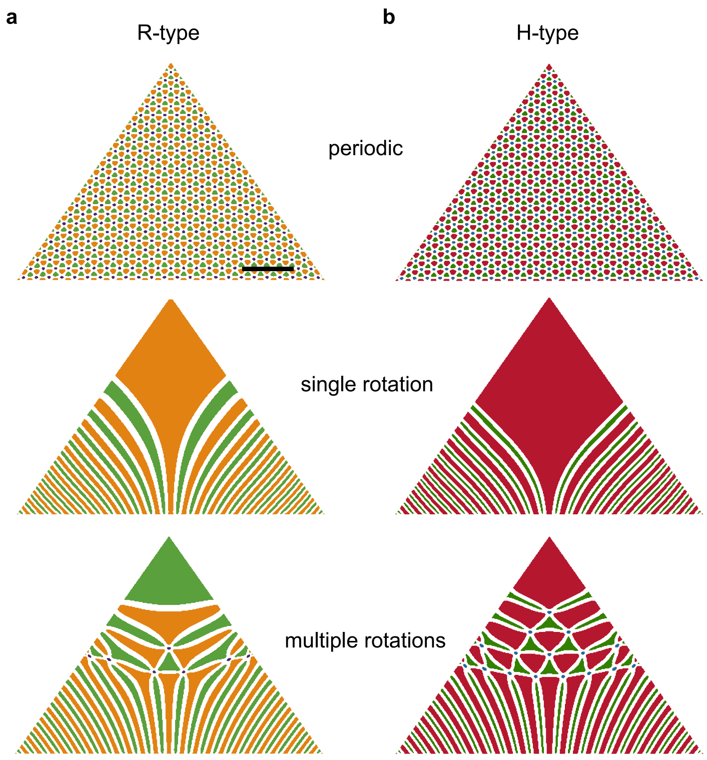

Supplementary Figure 6. **a** and **b**, Reconstruction patterns in the tips of triangular R- and H-type HBLs with $\theta = 3^\circ$ twist angle for one rotation point at $\alpha = 0$ (central panel) and two rotation points at $\alpha = 0$ and $\alpha = 1/12$ (bottom panel). The top maps show periodic reconstruction without rotation for reference. The scale bar is 25 nm.

Supplementary Note 3: Sample characteristics and spectral features

Experimental studies were mainly performed on four samples with $MoSe_2$-WSe_2 HBLs encapsulated in hBN with characteristics shown in Supplementary Fig. 7. Despite different geometries, all samples show two common features. First, aligned R-type HBLs are generally brighter than their H-type counterparts (as in sample 1 and 2 with nearly aligned R- and H-type HBLs), consistent with a factor of 10 difference in oscillator strengths of the lowest spin-singlet (R-type) and spin-triplet (H-type) interlayer exciton transitions. Second, all four samples exhibit substantial variations in PL intensity on micron scales across H- and R-type regions despite rather clean interfaces with only few interfacial bubbles. Moreover, spots of bright interlayer exciton PL are predominantly observed near HBL tips and edges, whereas sample cores are relatively dark. Based on observations in SEM and our reconstruction model, we interpret these spatial variations as arising from mesoscopic domain networks with extended 2D domains, 1D stripes and 0D domain arrays.

The spectral characteristics observed in cryogenic PL and differential reflectivity (DR) are shown in Supplementary Fig. 8 - 11 for positions representative of 2D domains, 1D stripes and 0D domain arrays in each sample. Note that the signatures of 1D stripes with a high degree of linear polarization were observed only in R-type HBLs (sample 1 and 2 in Supplementary Fig. 8 and Supplementary Fig. 9, respectively). Bright PL spots were consistently observed near line defects such as cracks and edges, whereas dark PL regions dominate the sample cores. In H-type samples, the PL characteristics of dark regions differ for nearly-aligned samples (samples 1, 2, and 4) and the sample with a twist angle of $\sim 3°$: Whereas the former exhibit quantum dot type features of localized excitons, the latter shows consistent exciton-polaron characteristics on different sample positions.

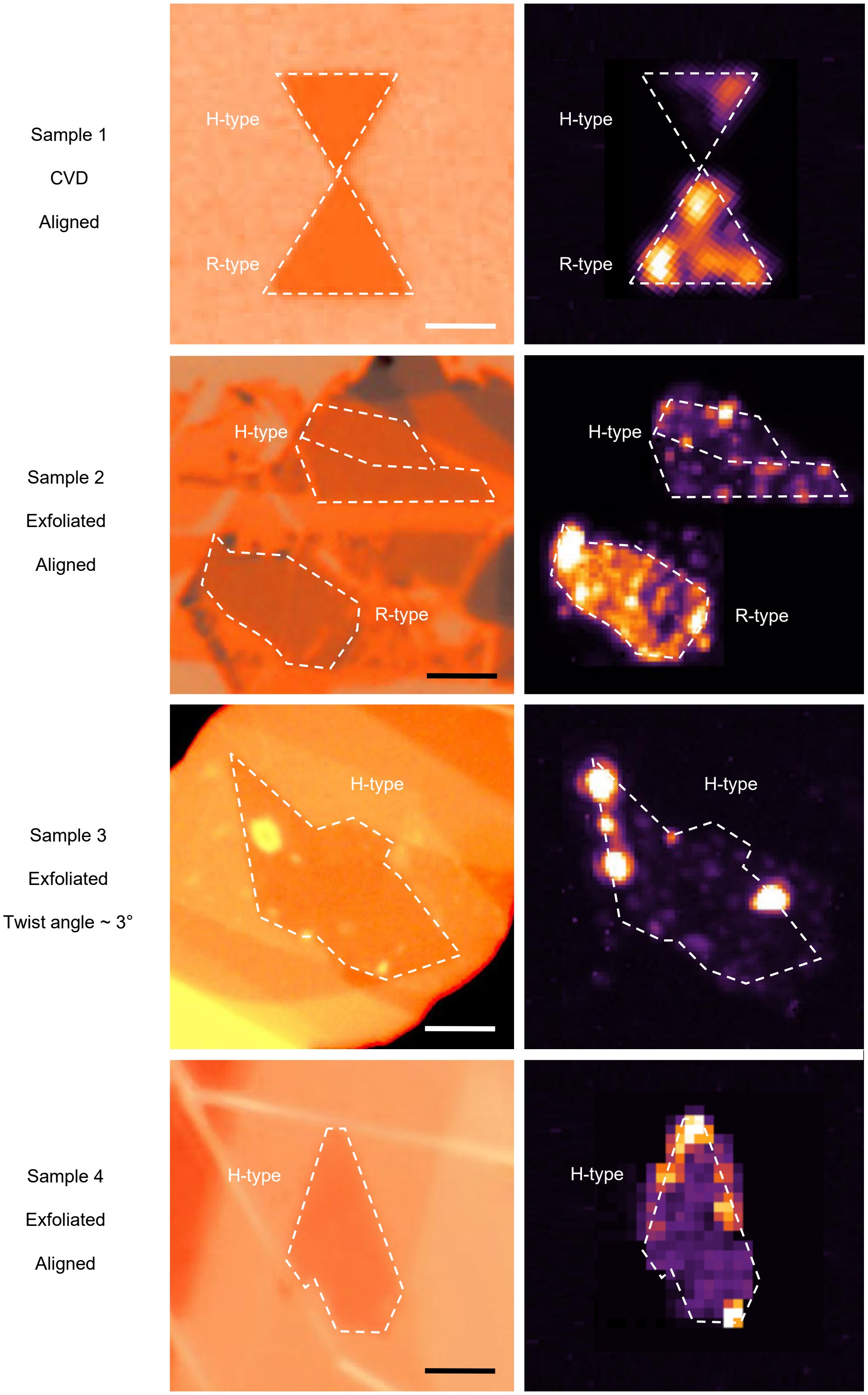

Supplementary Figure 7. Overview of hBN-encapsulated $MoSe_2$-WSe_2 HBL samples studied with optical spectroscopy (left panels: optical micrographs; right panels: raster-scan maps of PL intensity at 3.2 K within the interlayer exciton band $1.2 - 1.5$ eV excited with a laser at 725 nm and 2 μW excitation power). The scale bars are 4 and 2 μm for samples $1-3$ and sample 4, respectively.

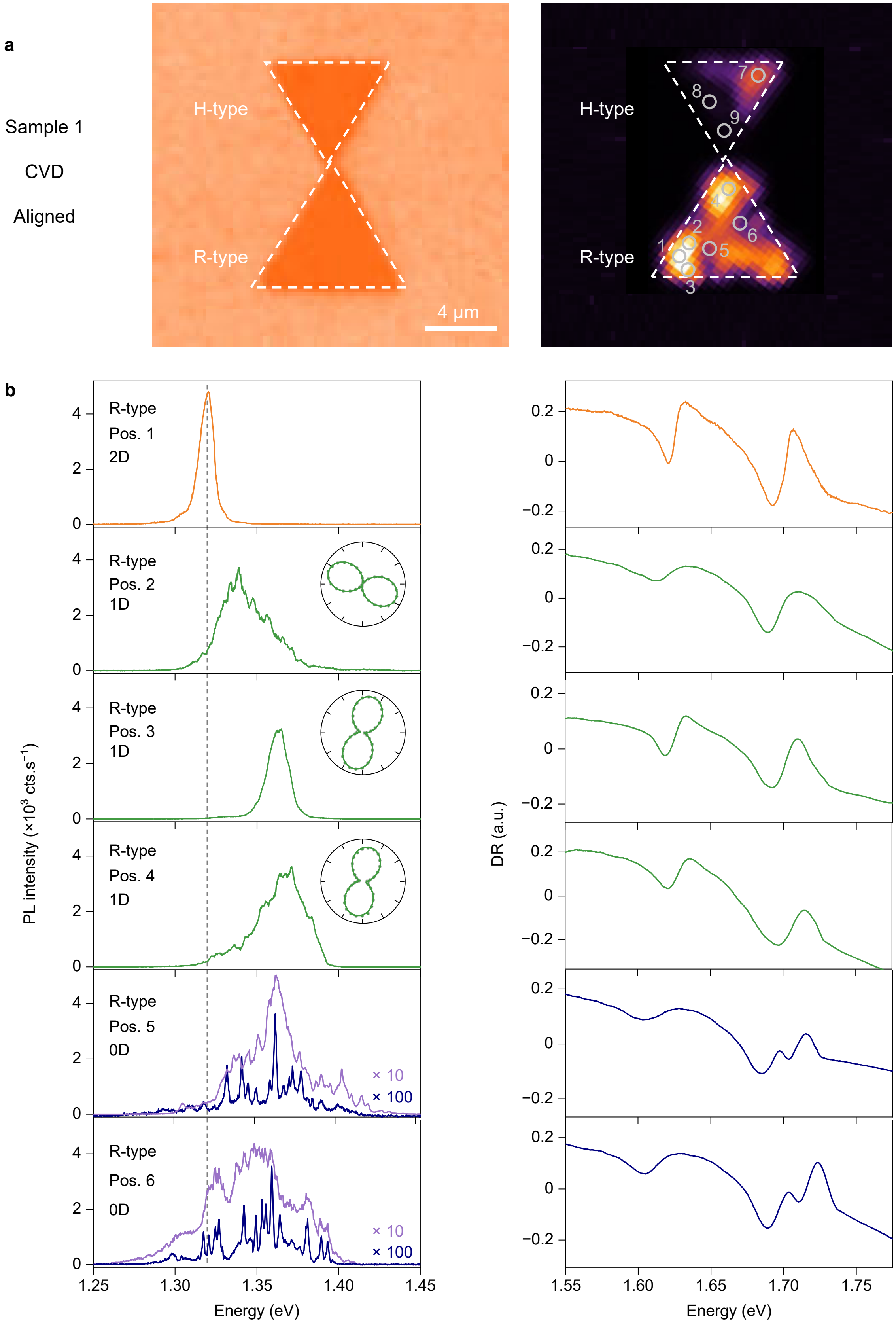
a
Sample 1
CVD
Aligned
H-type
R-type
4 μm
H-type
R-type
7
8
9
4
2
6
1
5
3
b
R-type
Pos. 1
2D
R-type
Pos. 2
1D
R-type
Pos. 3
1D
R-type
Pos. 4
1D
R-type
Pos. 5
0D
× 10
× 100
R-type
Pos. 6
0D
× 10
× 100
PL intensity (×10³ cts.s⁻¹)
Energy (eV)
DR (a.u.)
Energy (eV)

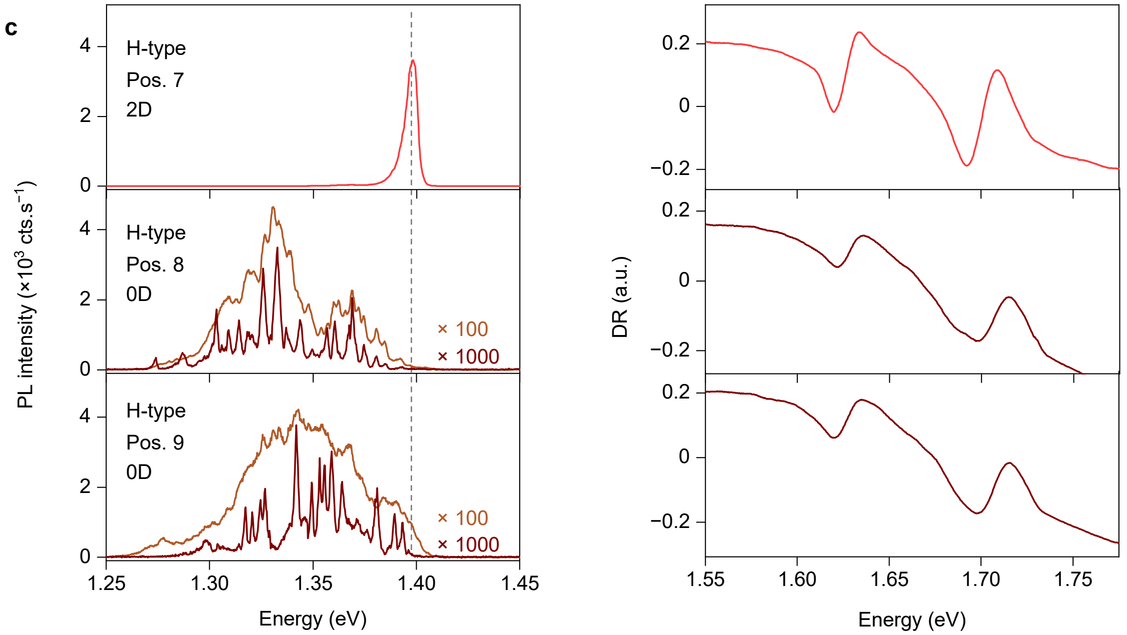

Supplementary Figure 8. Characteristics of sample 1. **a**, Left panel: optical micrograph; right panel: map of interlayer exciton PL intensity with selected positions for PL and DR spectra in **b** and **c**. **b**, PL (left panel) and DR (right panel) characteristics of R-type stack assigned to 2D domains (orange), 1D stripes (green) and 0D domain arrays (purple). **c**, PL (left panel) and DR (right panel) characteristics of H-type stack assigned to 2D domains (red) and 0D domain arrays (brown). The PL peak positions of 2D domains are shown by dashed lines for reference. For regions assigned to 1D stripes, the orientation of linearly polarized PL is specified by the insets. The PL spectra were recorded with an excitation power of 2 μW; for 0D arrays, additional spectra at 0.01 μW excitation power are shown (light and dark purple for R-type, light and dark brown for H-type) and scaled for better visibility (factors of 10 and 100 for R-type, 100 and 1000 for H-type).

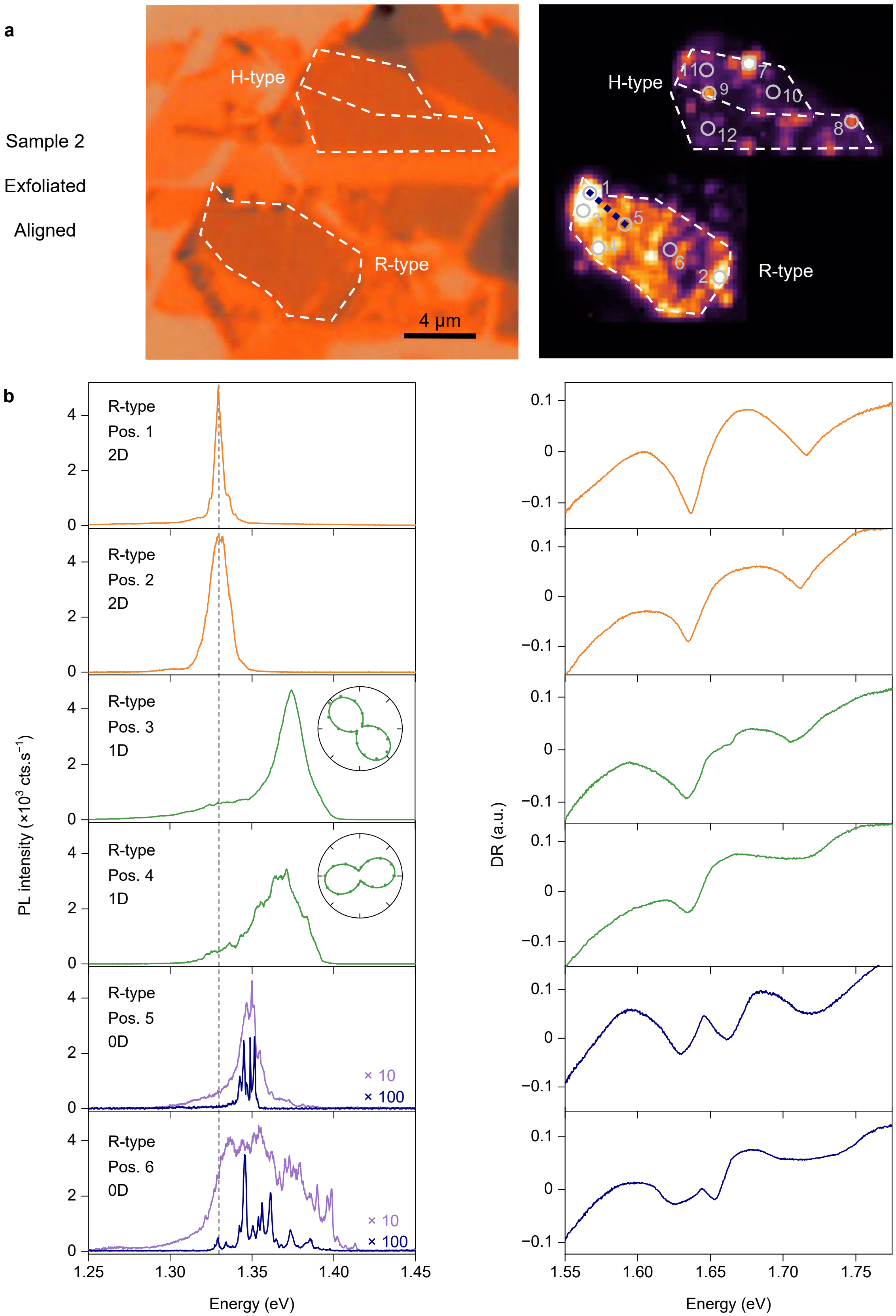

a
Sample 2
Exfoliated
Aligned
H-type
R-type
4 μm
b
R-type
Pos. 1
2D
Pos. 2
Pos. 3
1D
Pos. 4
Pos. 5
0D
Pos. 6
× 10
× 100
PL intensity (×10³ cts.s⁻¹)
DR (a.u.)
Energy (eV)

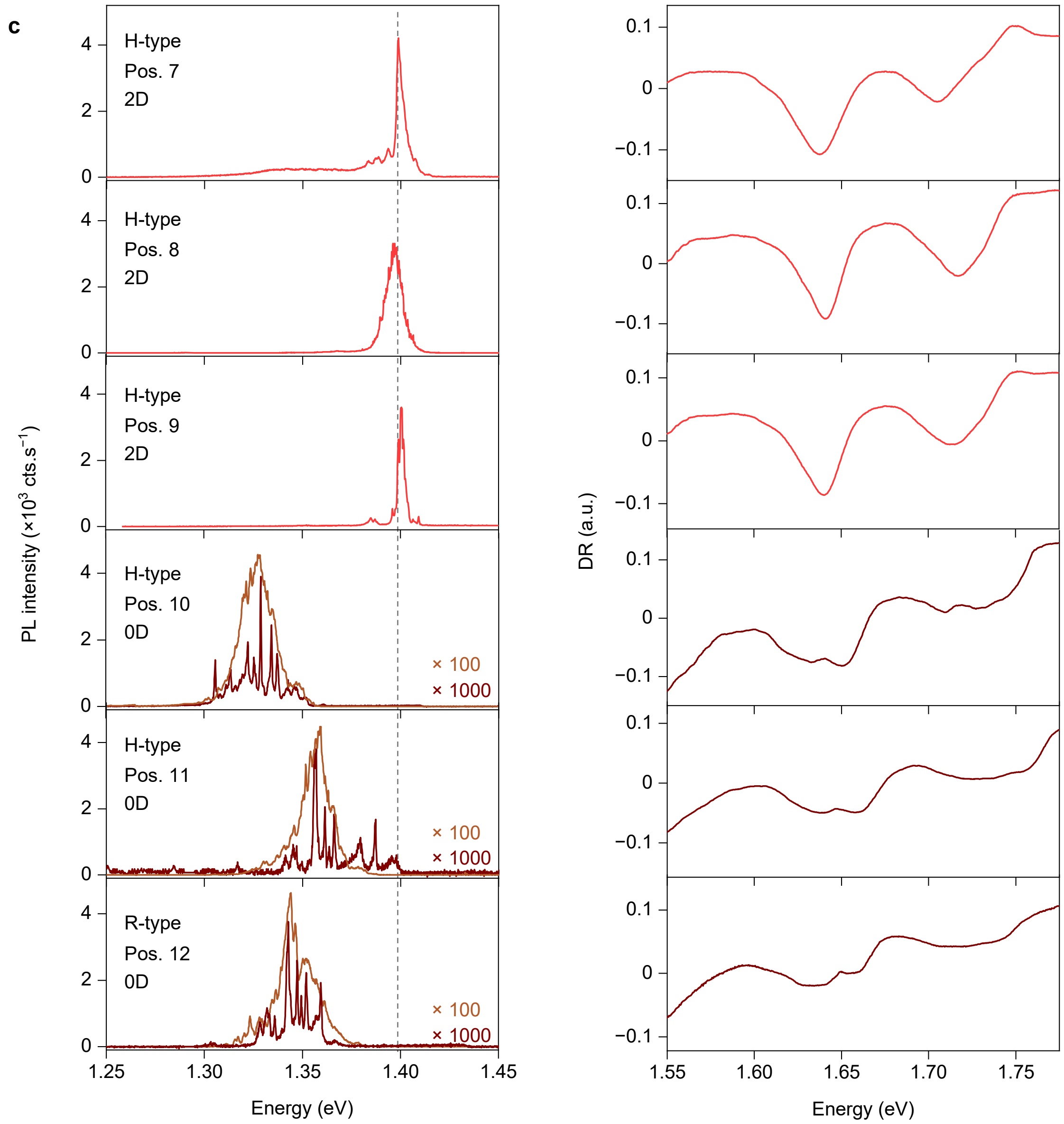

Supplementary Figure 9. Same as Supplementary Fig. 8 but for sample 2. The five black dots in the PL map in **a** indicate the corresponding positions of DR spectra in Fig. 3a of the main text.

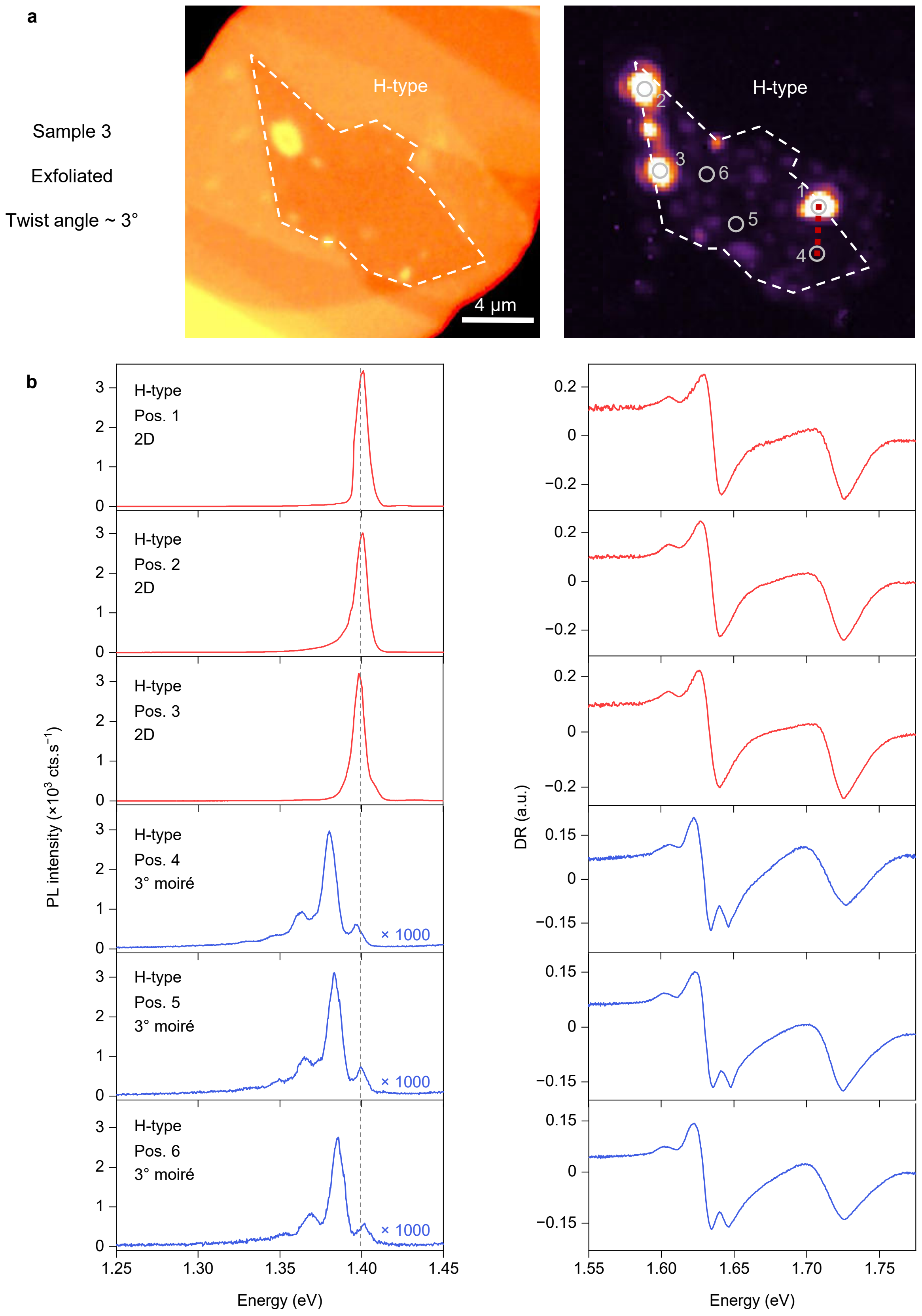

Supplementary Figure 10. Same as Supplementary Fig. 8 but for sample 3. Note the distinct PL features of exciton-polaron scaled by a factor of 1000. The five red dots in **a** indicate the positions of DR spectra in Fig. 4a of the main text.

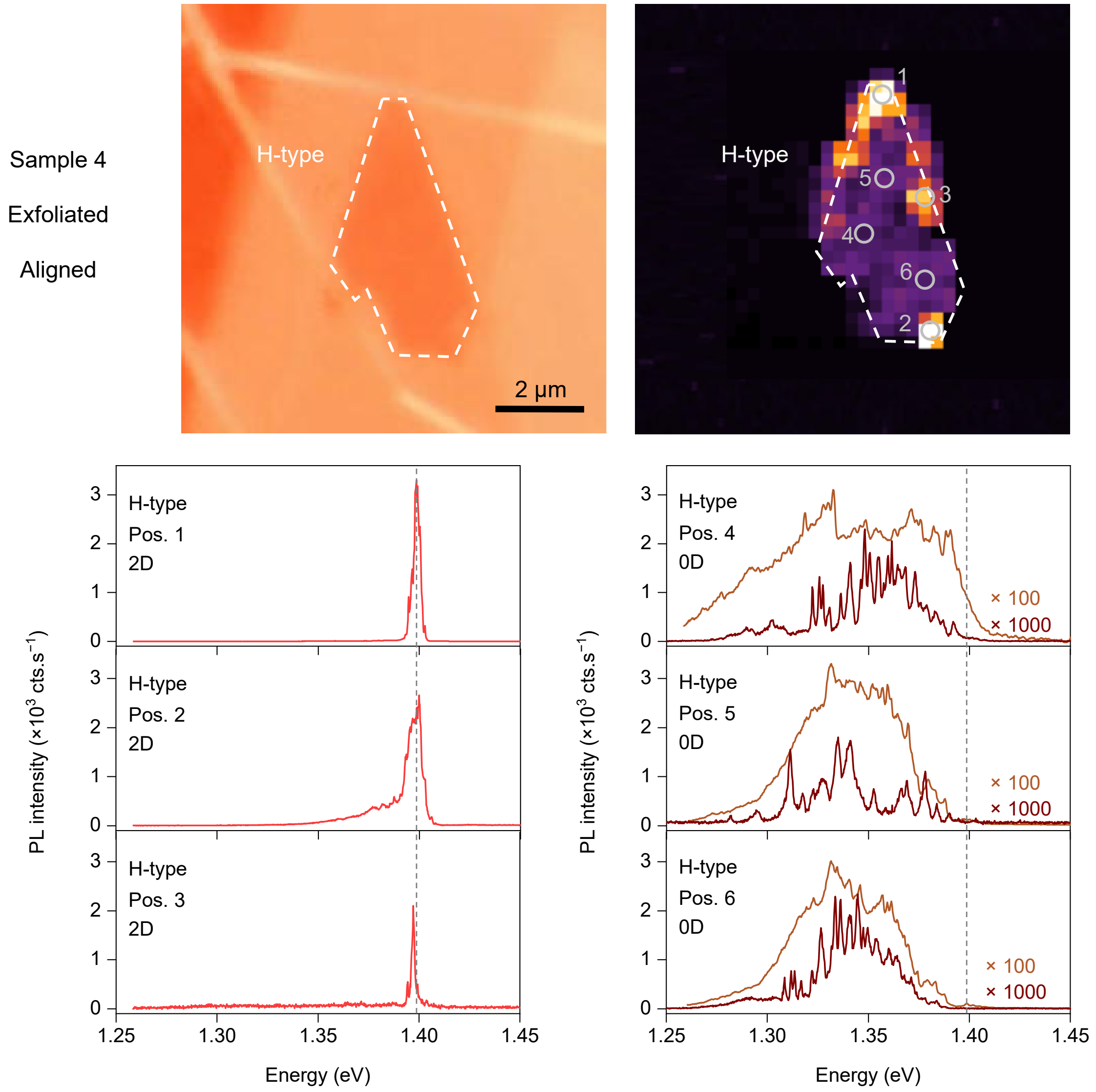

Supplementary Figure 11. Characteristics of sample 4 without DR data.

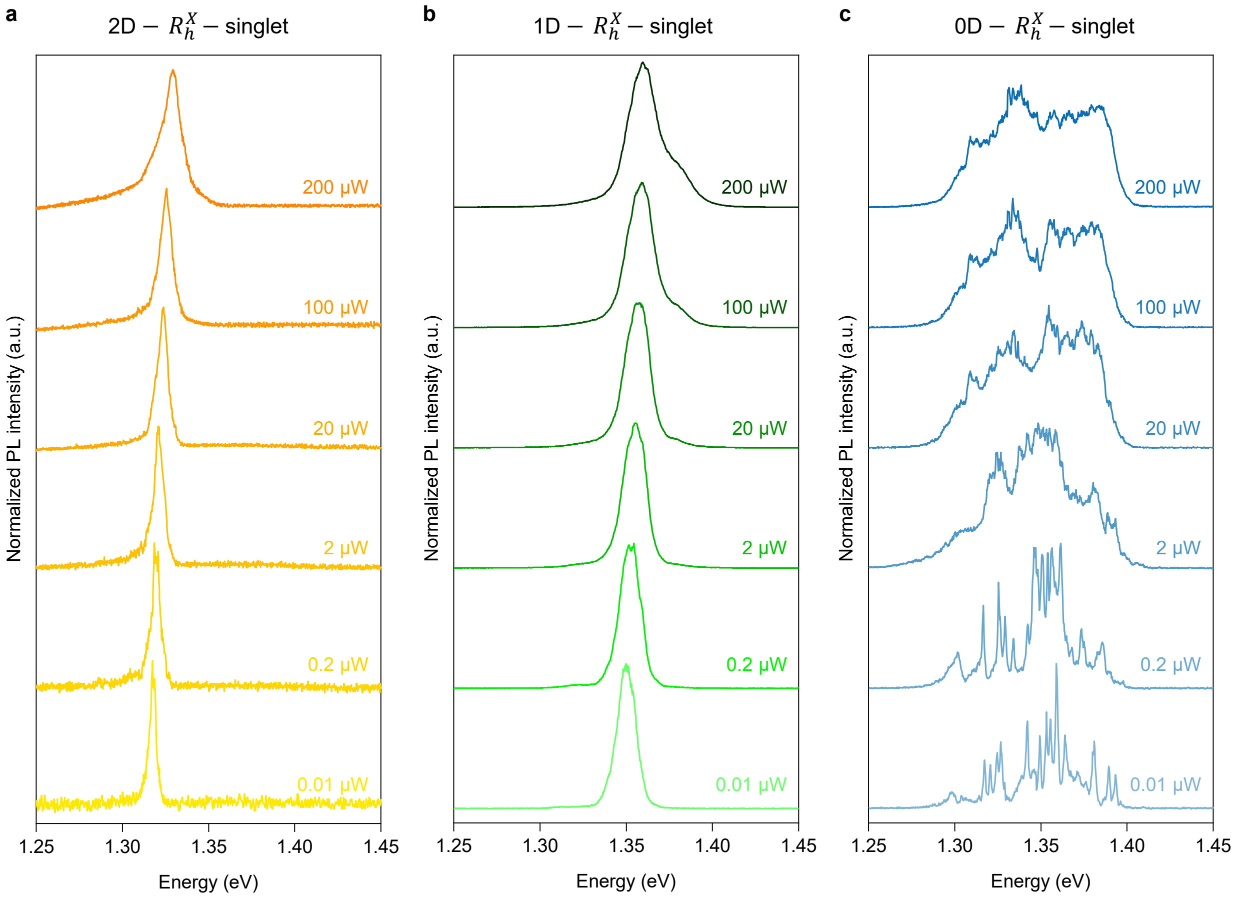

Supplementary Figure 12. Power-dependent interlayer PL of different domain types in nearly-aligned R-type HBL. **a** − **c**, Normalized PL spectra at selected excitation powers for a 2D domain (**a**), a region with 1D stripes (**b**), and domains of 0D arrays (**c**). All data were acquired on sample 1 with excitation at 725 nm.

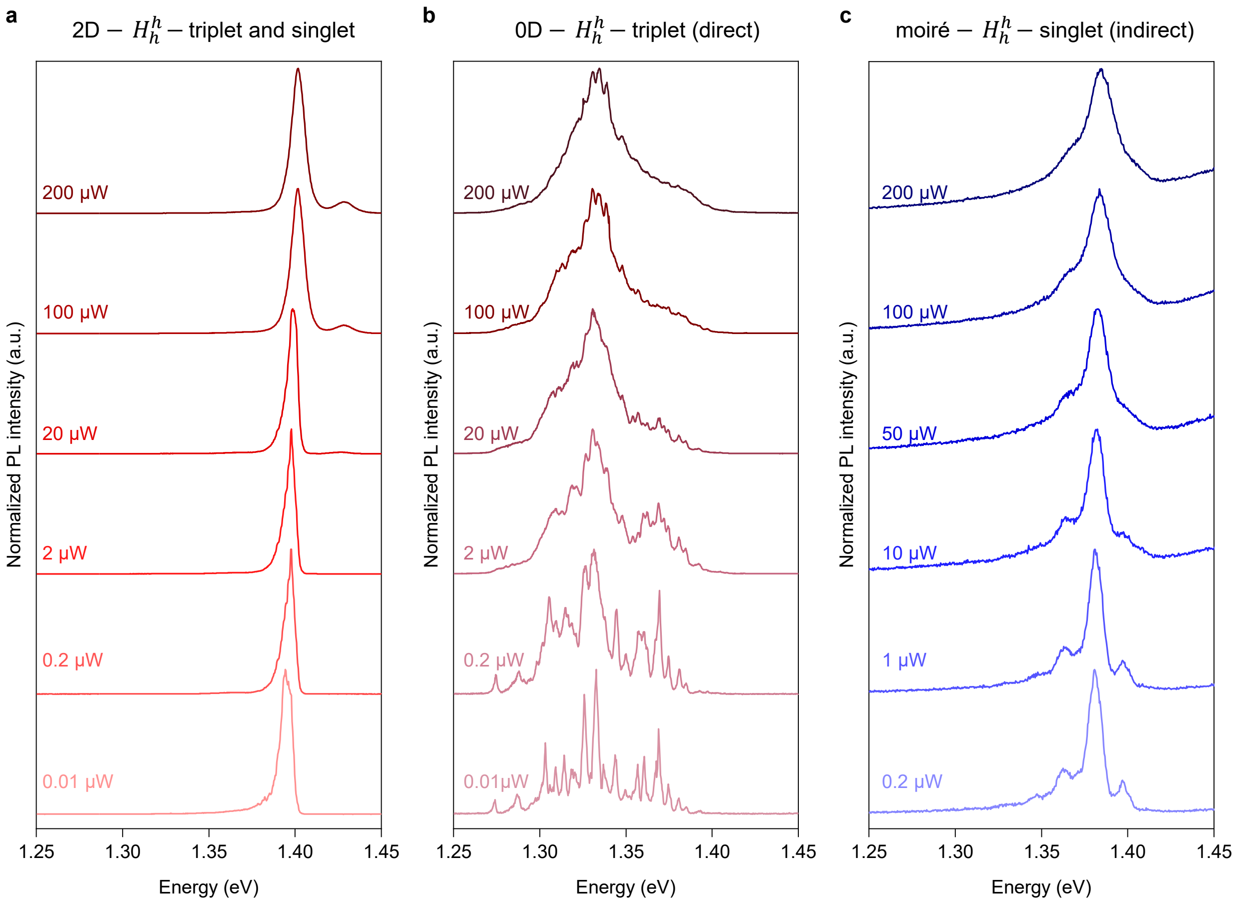

Supplementary Figure 13. Power-dependent interlayer PL of different domain types in H-type HBL. **a** − **c**, Normalized PL spectra at selected excitation powers for a 2D domain (**a**), domains of 0D arrays (**b**), and a dark region in a HBL moiré superlattice with 3° twist angle (**c**). The data in **a** and **b** were acquired on sample 1, the data in **c** are from sample 3. For all data, the excitation wavelength was 725 nm.

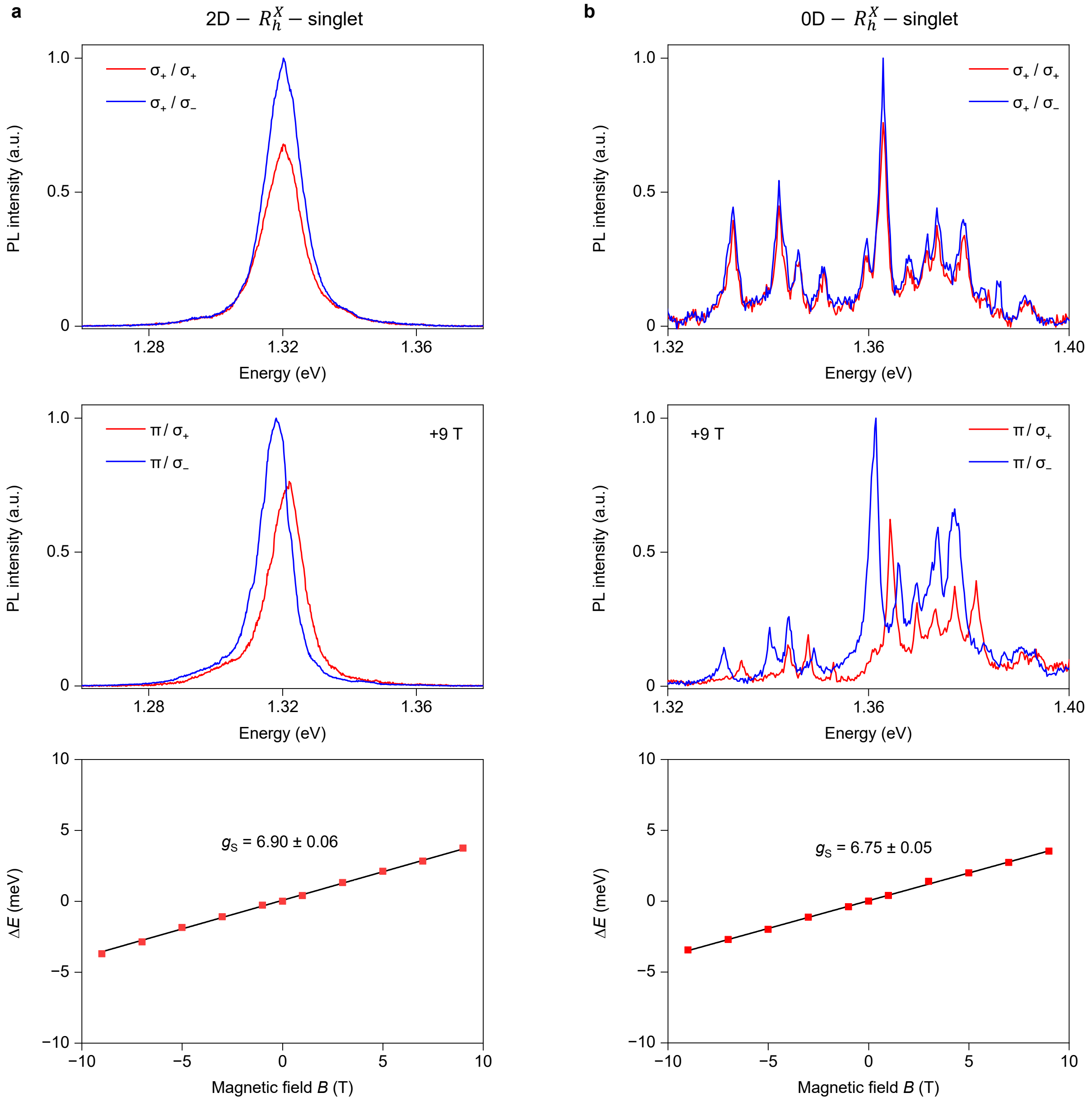

Supplementary Figure 14. Valley polarization and magneto-luminescence of interlayer excitons in nearly-aligned R-type HBL region of sample 1. with data in a 2D domain (**a**) and a 0D domain array (**b**). Top panels: Polarization-resolved PL (with σ_+ and σ_- polarization shown in red and blue) with σ_+ polarized excitation at 0 T. All peaks exhibit negative P_c. Central panels: Valley Zeeman splitting between σ_+ and σ_- polarized peaks in a magnetic field of 9 T under linearly polarized excitation (π). Bottom panels: Valley Zeeman splitting ΔE as a function of magnetic field. The solid lines are linear fits to the data with g-factors and error bars obtained from least-square best fits. The negative P_c and the positive g-factor assign the PL to singlet R_h^X interlayer exciton. All data were recorded with excitation powers of 20 and 0.01 μW for 2D and 0D regions.

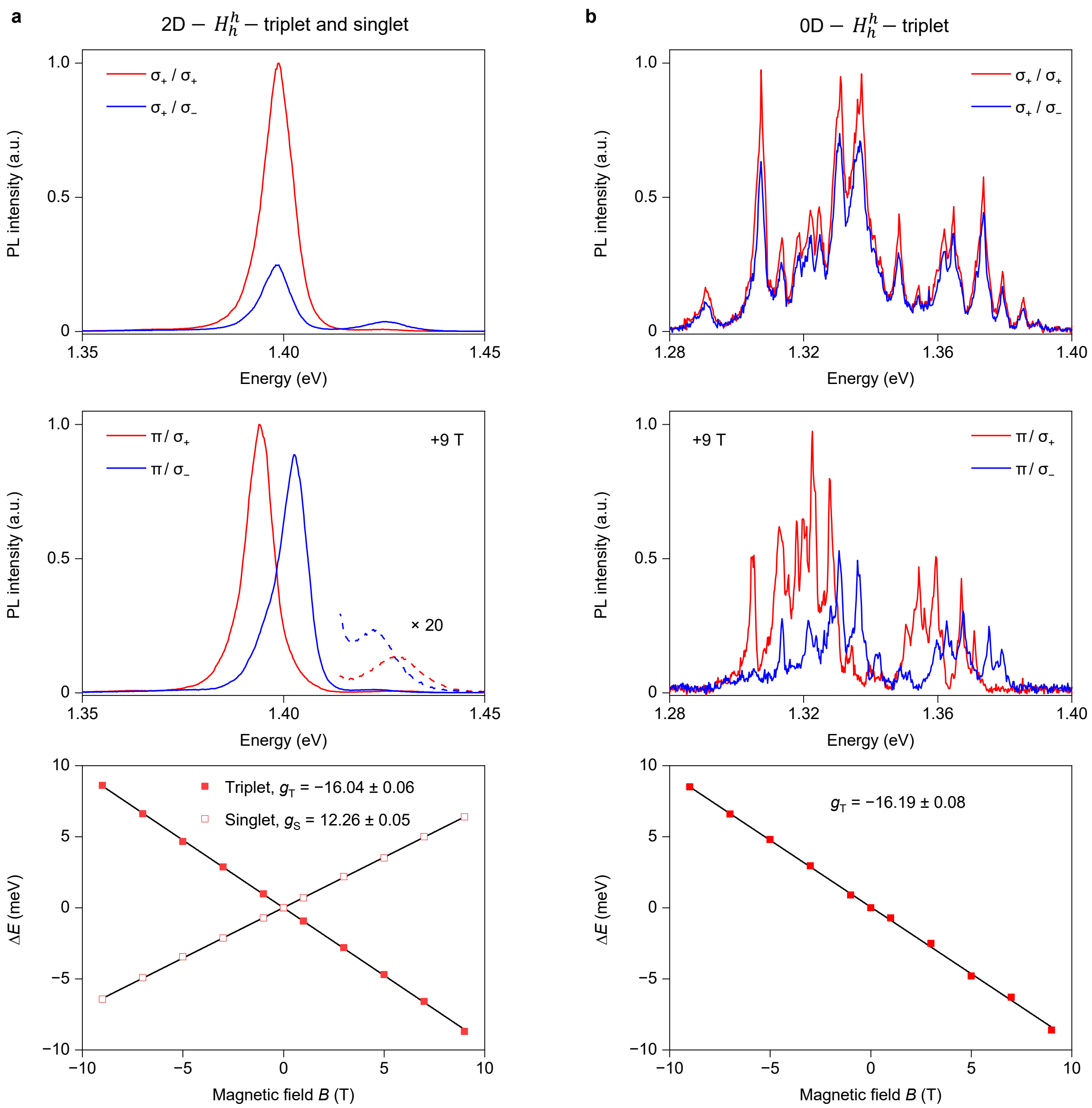

Supplementary Figure 15. Same as Supplementary Fig. 14 but for nearly-aligned H-type HBL in sample 1. All PL peaks exhibit positive P_c and g-factors of about -16, as obtained from linear fits to the data in the bottom panel with error bars from least-square best fits. Both features are characteristic of H_h^h triplet interlayer excitons. In the regions of 0D arrays, P_c is smaller than in extended 2D regions due to reduced symmetry of the interlayer exciton wavefunctions in domains with distorted hexagonal shapes. The weak peak in the spectra of panel **a** with 25 meV blue-shift from the main peak corresponds to H_h^h singlet interlayer excitons with negative P_c and positive g-factor of ~ 12.

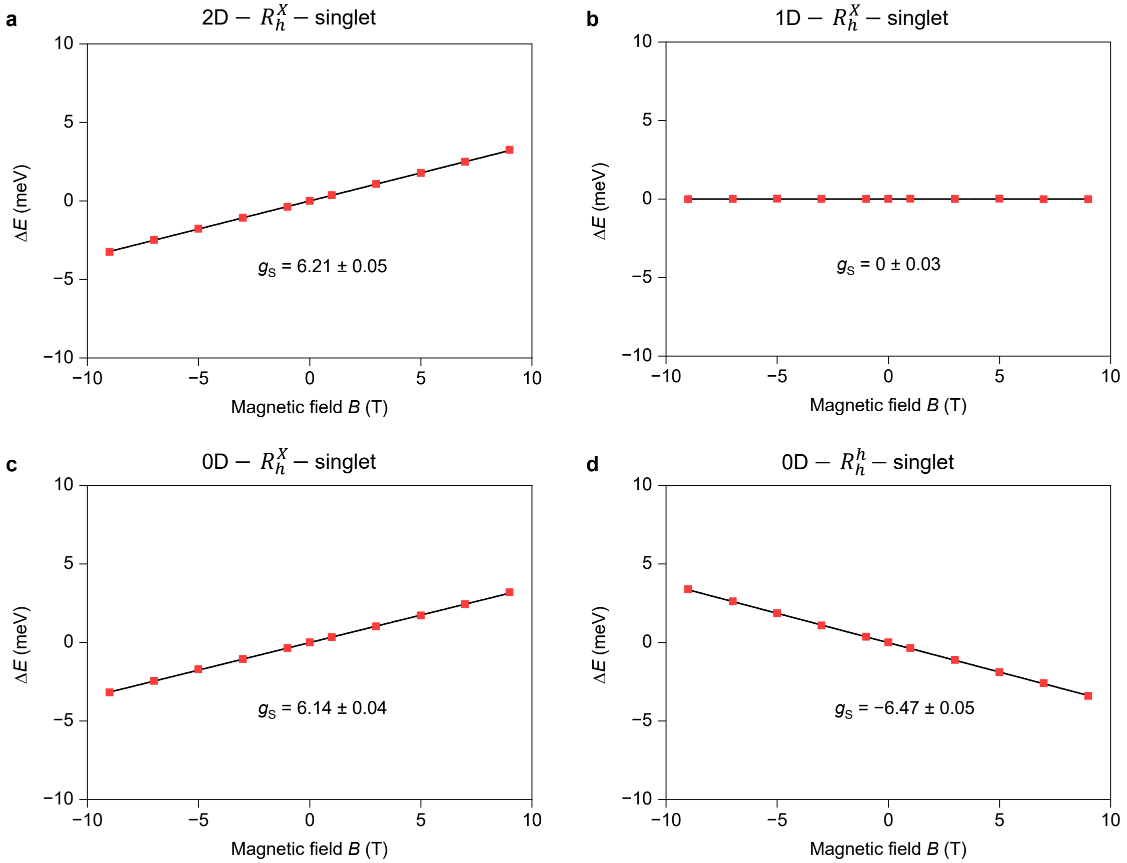

Supplementary Figure 16. Magneto-luminescence of interlayer excitons in nearly-aligned R-type HBL region of sample 2: valley Zeeman splitting ΔE as a function of magnetic field obtained as the energy difference between σ_+ and σ_- polarized peaks of interlayer exciton PL under linearly polarized excitation. The solid lines are linear fits to the data with g-factors and error bars obtained from least-square best fits. The data in **a** and **b** correspond to the data in false-color representation in Fig. 3**d** and **g** of the main text; the data in **c** and **d** correspond to the data in Fig. 3**j** of the main text. All data were recorded on sample 2.

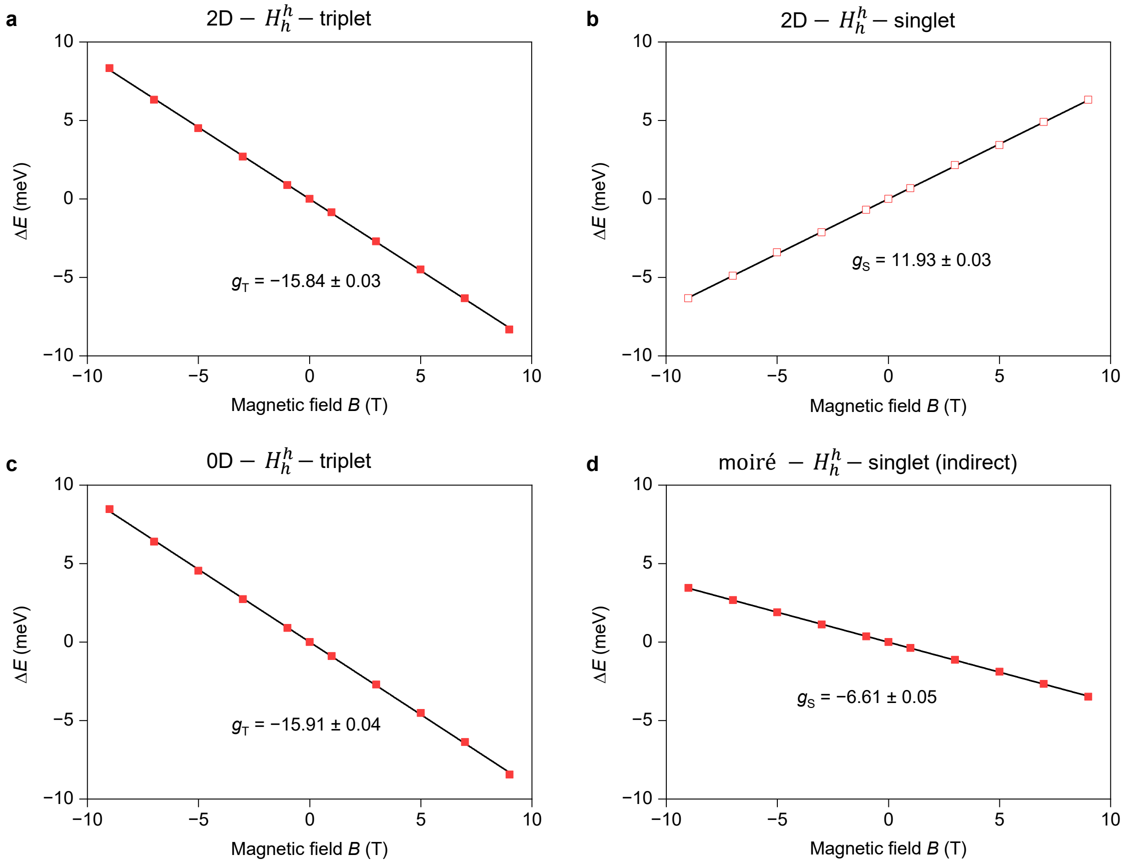

Supplementary Figure 17. Magneto-luminescence of interlayer excitons in H-type HBL of sample 2 and sample 3: valley Zeeman splitting ΔE as a function of magnetic field obtained as the energy difference between σ_+ and σ_- polarized peaks of interlayer exciton PL under linearly polarized excitation. The solid lines are linear fits to the data with g-factors and error bars obtained from least-square best fits. The data in **a** and **b** correspond to the data in false-color representation in Fig. 4**d** of the main text; the data in **c** and **d** correspond to the data in Fig. 4**g** and **j** of the main text, respectively. Data in **a**, **b** and **c** were recorded on sample 2, and data in **d** are from sample 3.

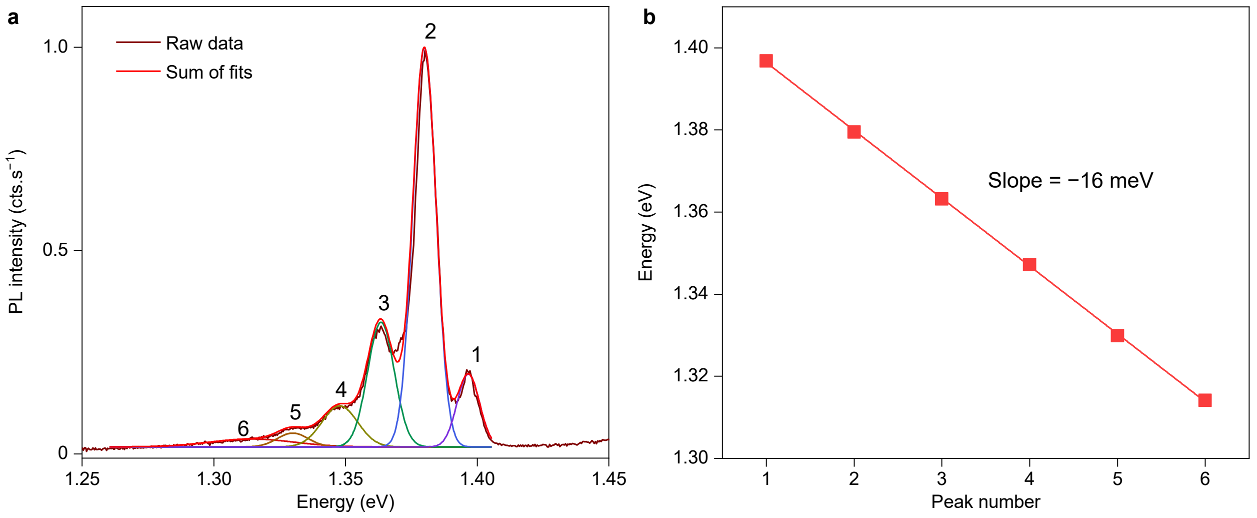

Supplementary Figure 18. **a**, Representative PL spectrum from a dark region in H-type HBL moiré superlattice with 3° twist angle (sample 3). In contrast to 0D arrays of nearly-aligned samples, the spectrum is characterized by a series of six peaks (shown together with Gaussian fits). **b**, Energy of six Gaussian peaks in **a** plotted as a function of the peak number. The linear fit indicates equidistant peak spacing of 16 meV.

Supplementary Note 4: Time-resolved photoluminescence of reconstructed domains

The PL decay of interlayer excitons in different domain types was studied with time-resolved PL spectroscopy, with main results for sample 1 shown in Supplementary Fig. 19 and Supplementary Table 1 for an excitation power of 0.05 μW at 725 nm excitation wavelength. The set of data is complemented by power-dependent lifetime measurements of R_h^X singlet and H_h^h triplet interlayer excitons in Supplementary Fig. 20. Focusing first on reconstructed 2D domains, where interlayer exciton reservoirs are limited to zero-momentum and momentum-dark exciton states in H_h^h and R_h^X registries due to the absence of other stackings, we determine from best-fit analysis (taking into account the instrument response function) two decay channels for the H_h^h singlet exciton and three decay channels for both the H_h^h triplet and R_h^X singlet excitons. According to Supplementary Table 1, the H_h^h singlet PL is characterized by two decay times - one below the resolution limit of 0.2 ns and one with a decay time of 2.5 ns. We ascribe the primary decay time to population loss of H_h^h singlet excitons by rapid relaxation into the energetically lower reservoir of H_h^h triplet excitons and other non-radiative decay processes including Auger-mediated population loss.

The secondary decay channel with 2.5 ns decay time reflects the characteristic lifetime of H_h^h singlet interlayer exciton state which is proportional to the oscillator strength of the respective radiative transition.

Using 2.5 ns as the radiative lifetime of the H_h^h singlet exciton reservoir, we estimate by scaling of oscillator strengths from Table 1 of the main text the radiative PL lifetimes of 65 ns for the H_h^h triplet interlayer exciton reservoir, as well as 5.3 and 23 ns for the R_h^X singlet and triplet exciton reservoirs, respectively. Experimentally, the secondary decay component of the H_h^h triplet reservoir and the R_h^X singlet reservoir are determined to 48 ns and 5 ns, respectively, in good agreement with the scaling anticipated from theory, whereas the PL from the R_h^X triplet state is insufficient for time-resolved measurements. The contribution of Auger decay to the primary decay channel is evident for the H_h^h triplet and R_h^X singlet PL decay from power-dependent data in Supplementary Fig. 20, where the amplitude of the primary component dominates at high excitation powers over the amplitudes of the two complementary channels with power-independent ratio.

With this assignment of primary and secondary PL decay components to non-radiative population loss processes (including Auger) and radiative decay, respectively, we attribute the third channel of H_h^h triplet exciton PL decay on 440 ns timescale and R_h^X singlet exciton state with 45 ns decay time to population feeding from energetically higher-lying long-lived reservoirs, presumably constituted by momentum-dark configurations of valence band holes in WSe_2 and conduction band electrons in $MoSe_2$. Note that the absence of such reservoirs could explain the lack of the third channel in the PL decay of the H_h^h singlet state that is the top-most energy level according to our calculations summarized in Table 1 of the main text.

For domains with reduced dimensionality, we observe similar behavior in time-resolved PL. With increasing quantum confinement effects due to reduced system dimensionality, R_h^X states show the trend of prolonged lifetimes for 1D stripe and 0D array regions. The overall ratio of the three components shows no strong dependence on dimensionality. The trend is opposite in 0D arrays of H_h^h triplet excitons, where all decay components decrease as compared to the 2D domain limit, consistent with the absence of quantum confinement effects in reconstructed domains of H_h^h stacking and potentially related to population loss into surrounding H_h^M and H_h^X domain walls with lower-energy interlayer exciton states.

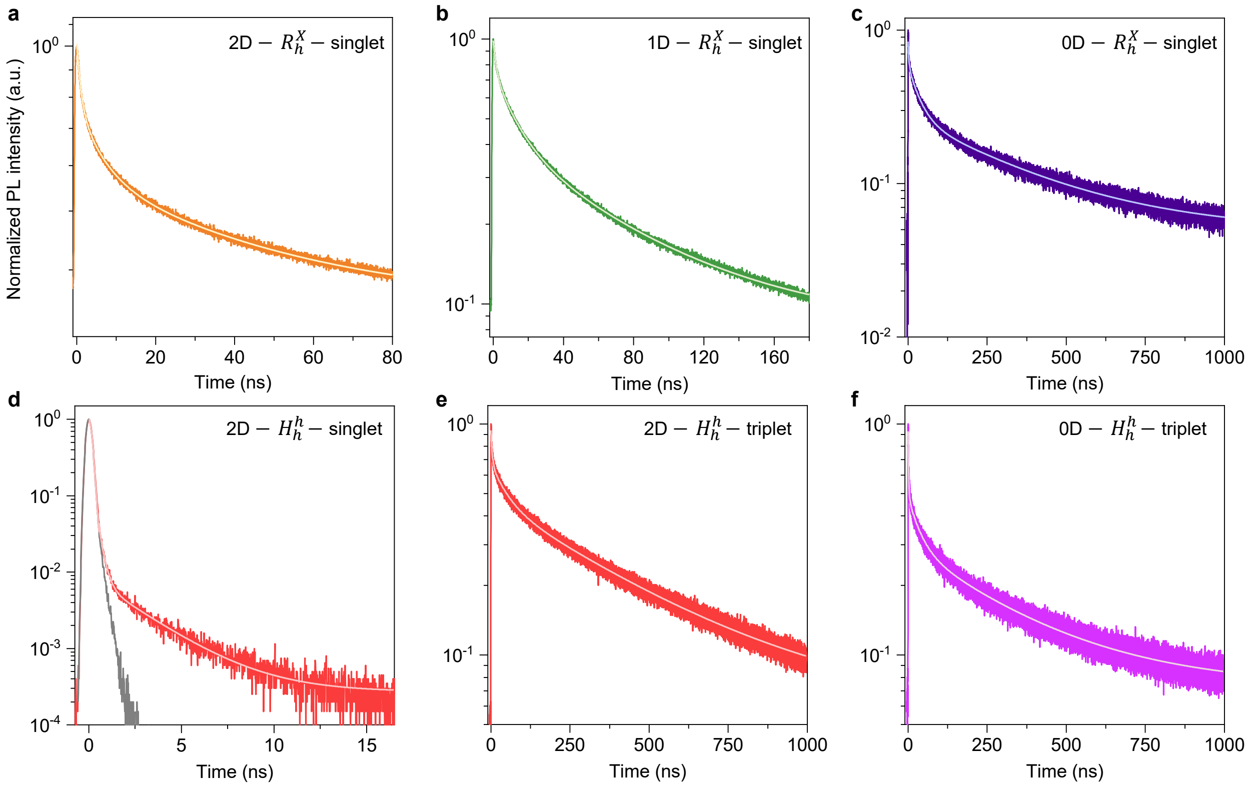

Supplementary Figure 19. Time-resolved PL of interlayer excitons in different types of domain. **a** – **c**, Time-resolved PL decay of R_h^X singlet exciton in a 2D domain (**a**) and regions of 1D stripes (**b**) and 0D arrays (**c**). **d** – **f**, Time-resolved PL decay of H_h^h singlet exciton (**d**) and triplet exciton (**e**) in a 2D domain region, as well as H_h^h triplet exciton in a 0D domain region (**f**). The instrument response function (IRF) is shown in **d** in gray. The solid lines show in each panel best fits to the data obtained by a convolution of the IRF and a tri-exponential decay. The best-fit parameters are listed in Supplementary Table 1. All data were recorded on sample 1 with excitation at 725 nm and 0.05 μW power.

Domain	Stacking	Spin configuration	τ_1	A_1	τ_2	A_2	τ_3	A_3
2D	R_h^X	singlet	1.1 ns	34%	5 ns	30%	45 ns	36%
1D	R_h^X	singlet	1.5 ns	25%	13 ns	37%	70 ns	38%
0D	R_h^X	singlet	2.2 ns	32%	34 ns	40%	335 ns	28%
2D	H_h^h	singlet	$<$ 0.2 ns	98%	2.5 ns	2%		
2D	H_h^h	triplet	2.4 ns	29%	48 ns	24%	440 ns	47%
0D	H_h^h	triplet	1.7 ns	52%	29 ns	22%	305 ns	26%

Supplementary Table 1. Best-fit parameters obtained for the data in Supplementary Fig. 19 using tri-exponential decay with characteristic lifetime (τ_i) and amplitude (A_i) of each decay channel.

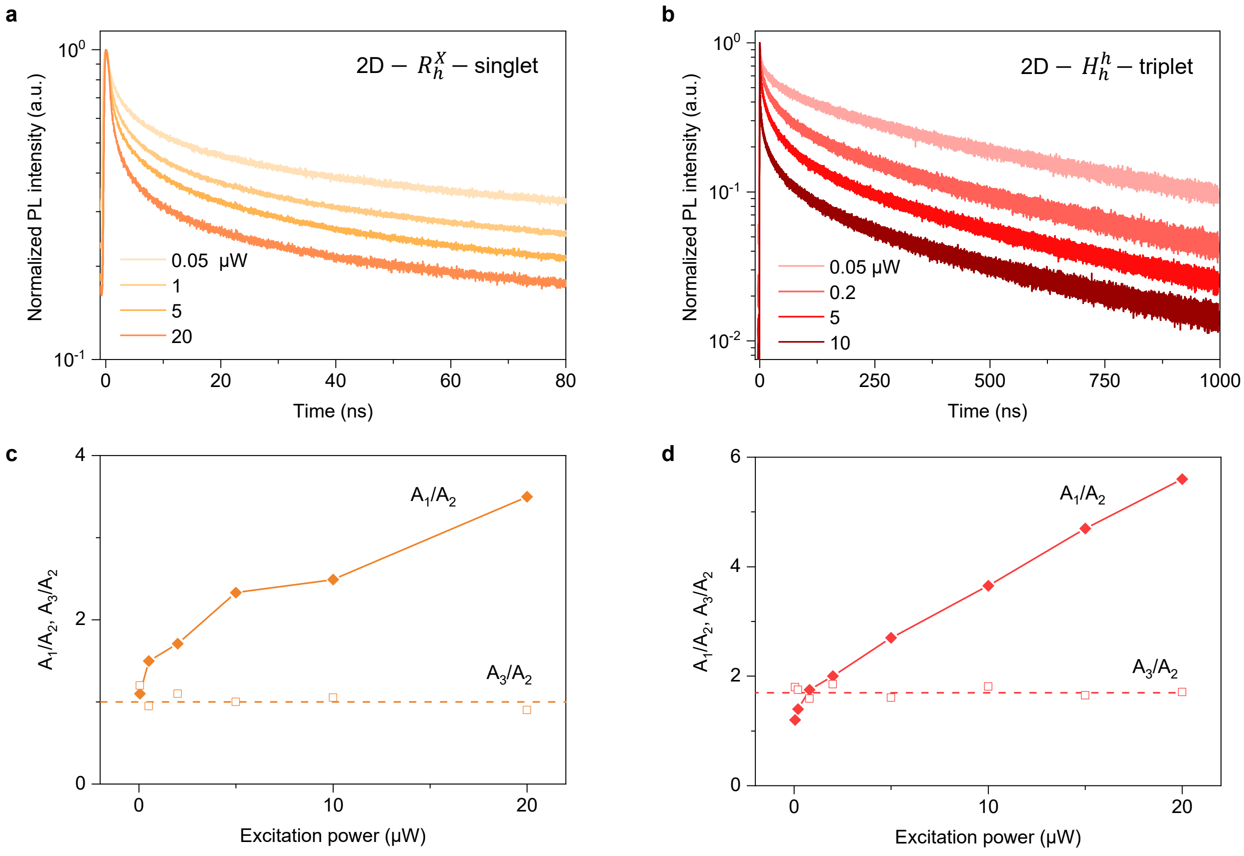

Supplementary Figure 20. Power dependent PL decay. **a** and **b**, Time-resolved PL decay of R_h^X singlet (**a**) and H_h^h triplet (**b**) interlayer exciton in 2D domains for different excitation powers. **c** and **d**, The amplitude ratios between the fast and intermediate decay components (A_1/A_2, shown by filled diamonds) and between the intermediate and long decay components (A_2/A_3, shown by empty squares) as a function of excitation power. According to best-fits, the three characteristic decay times are independent of the excitation power. However, the fast decay component becomes dominant at high excitation powers as A_1/A_2 increases but A_2/A_3 remains constant, ascribing the fast decay channel to Auger-mediated processes.

Supplementary Note 5: Direct correlation between domain patterns and optical properties

To provide a direct evidence for correlations between mesoscopically reconstructed domain dimensionality and respective optical features, we performed SEM imaging and cryogenic optical spectroscopy on the same $MoSe_2$-WSe_2 HBL. To this end, we fabricated one H-type and one R-type fully hBN encapsulated samples with thin hBN top layers below 5 nm to ensure sufficient transparency for secondary electrons. Since the thin hBN layer is too fragile to pick up additional TMD and hBN layers of the heterostructure, the sample fabrication process was modified. We first picked up a thick hBN flake with the PC/PDMS stamp. This hBN layer was then used to pick up successively $MoSe_2$ and WSe_2 monolayers and a thin hBN flake. The PC film together with the stacked sample was subsequently cut off from the PDMS stamp, flipped over and transferred onto a 300 nm SiO_2/Si chip. After high vacuum annealing at 450° for 2 h, the wafer was carefully immersed in chloroform for 5 min to remove the PC film. With this procedure, we obtained a clean heterostack with the thin hBN layer on top.

Supplementary Fig. 21 **a** and **b** show the secondary electron modulated SEM image and interlayer exciton PL intensity map of the H-type heterostack. The SEM image shows coexisting 2D, 1D and 0D domains. Consistently, the PL map exhibits sizable intensity variations across the sample. By co-aligning the PL map and the SEM image (Supplementary Fig. 21 **c**), we correlate the spectral features with the local characteristics of domain geometry. All observations and conclusions from direct correlation between reconstructed domain structures and optical properties are consistent with the analysis provided in the main text.

For large 2D domains, exemplified in Supplementary Fig. 22 **a** and **b**, the interlayer PL is bright and characterized by a single peak at ∼1.40 eV of the triplet exciton state in H_h^h stacking. Consistently, the corresponding DR spectra feature single-peak resonances of intralayer excitons in $MoSe_2$ and WSe_2. In these domains, the degree of circular polarization is high and the degree of linear polarization is zero, as indicated by numbers in Supplementary Fig. 23 **a** and **b**.

For regions with elongated 1D domains, exemplified in Supplementary Fig. 22 **c** and **d**, the interlayer exciton PL is detected around 1.40 eV or with slightly red-shifted additional peaks. In contrast to 1D stripes in R-type HBLs, the PL preserves a sizable degree of

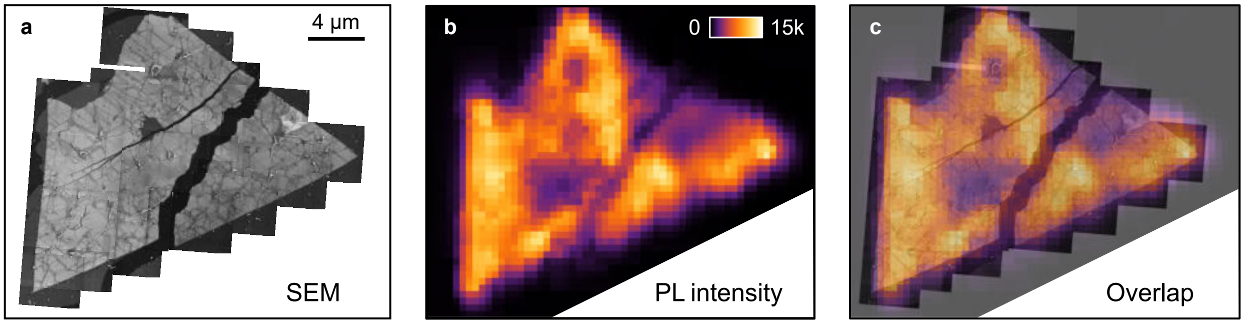

Supplementary Figure 21. **a**, Scanning electron micrograph of the H-type sample, composed of individual small-area images, with characteristic features of sample imperfections and mesoscopic reconstruction. **b**, PL intensity map in the spectral band of interlayer excitons at 3.2 K. **c**, Overlay of the SEM image in **a** and the PL map in **b**.

circular polarization, whereas the degree of linear polarization is either negligible or very small where the density of 1D stripes is high (Supplementary Fig. 23 **c** and **d**). As argued in the main text, pronounced effects of 1D quantum confinement are expected in R-type stacks where R_h^M domains represent potential wells for R_h^X exciton states. In H-type stacks, on the contrary, H_h^h excitons are energetically highest and thus deprived of efficient quantum confinement by surrounding domains of alternative registries. In these regions, structured PL and broadened DR spectra reflect variations in the size of 1D domains.

For 0D array regions, finally, exemplified in Supplementary Fig. 22 **e** and **f**, the interlayer exciton PL is very low and red-shifted by several tens of meV compared to 2D domains. This red-shift is size-dependent, with smaller domains exhibiting larger red-shifts. Moreover, for excitation powers below 100 nW, the PL spectra develop into quantum dot like spectrally narrow peaks. As shown in Supplementary Fig. 23 **e** and **f**, the degree of linear polarization is zero in the regions of 0D domains, and the degree of circular polarization is reduced as compared to extended 2D domain regions because of lower symmetry of the interlayer exciton wave functions in the presence of distorted hexagonal lattice of 0D arrays. The corresponding DR spectra exhibit for both $MoSe_2$ and WSe_2 intralayer exciton resonances characteristic splittings and broadenings which depend on the local homogeneity of domain sizes and shapes. Smaller domains feature larger energy splittings in the DR spectra, consistent with the analysis in the subsequent Supplementary Note 6.

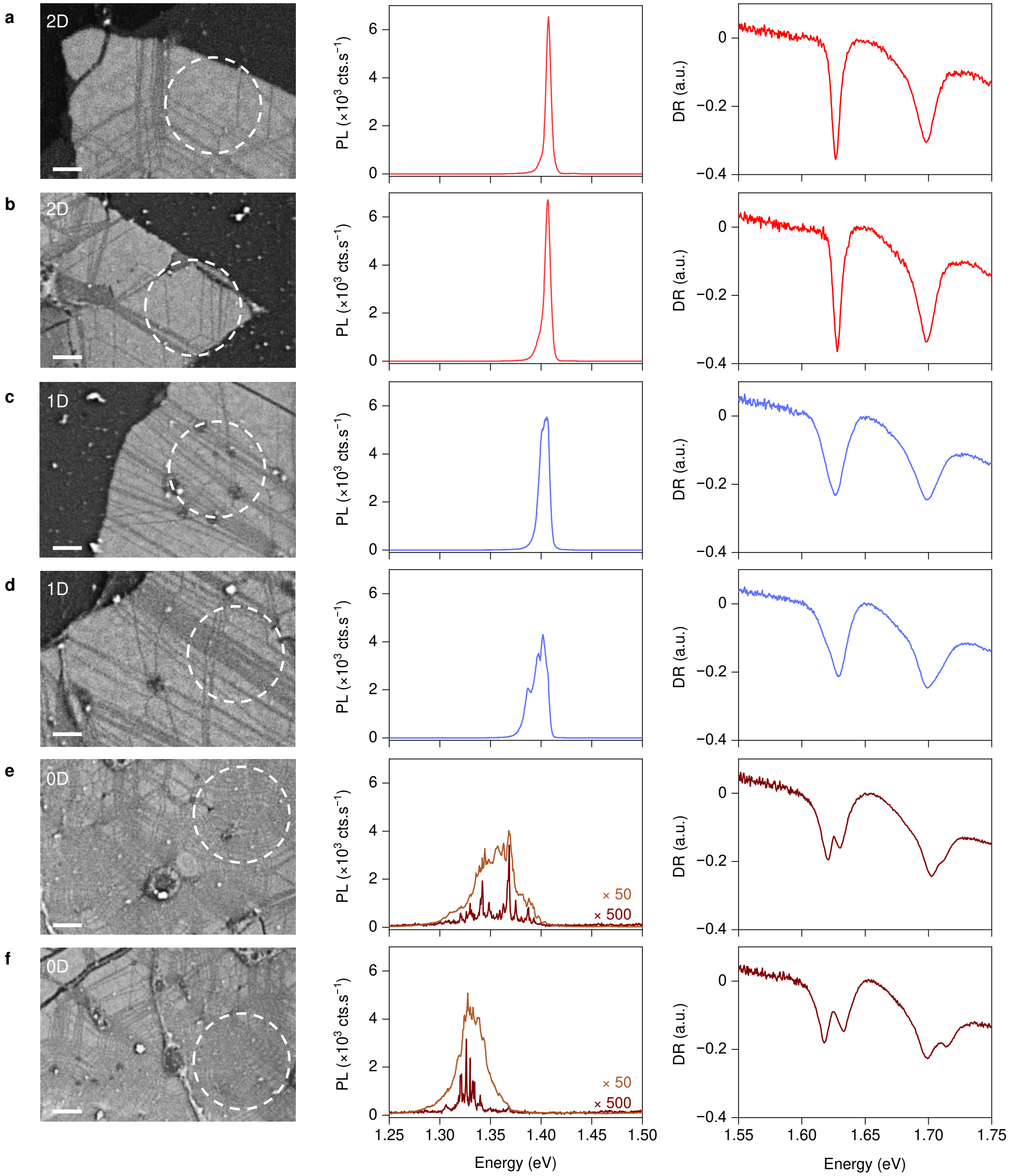

Supplementary Figure 22. Examples of reconstruction patterns and the corresponding optical spectra in the H-type sample. **a** – **f**, Left panels show representative areas of 2D (**a** and **b**), 1D (**c** and **d**) and 0D (**e** and **f**) domains identified in SEM imaging (the scale bars are 200 nm). Central and right panels show the corresponding PL and DR spectra within the optical spot delimited by dashed circles. The PL spectra were excited with 2 μW laser power. For 0D domains, the PL spectra at 0.01 μW are shown (dark brown) with respective scaling factors in addition to the spectra recorded at 2 μW (light brown).

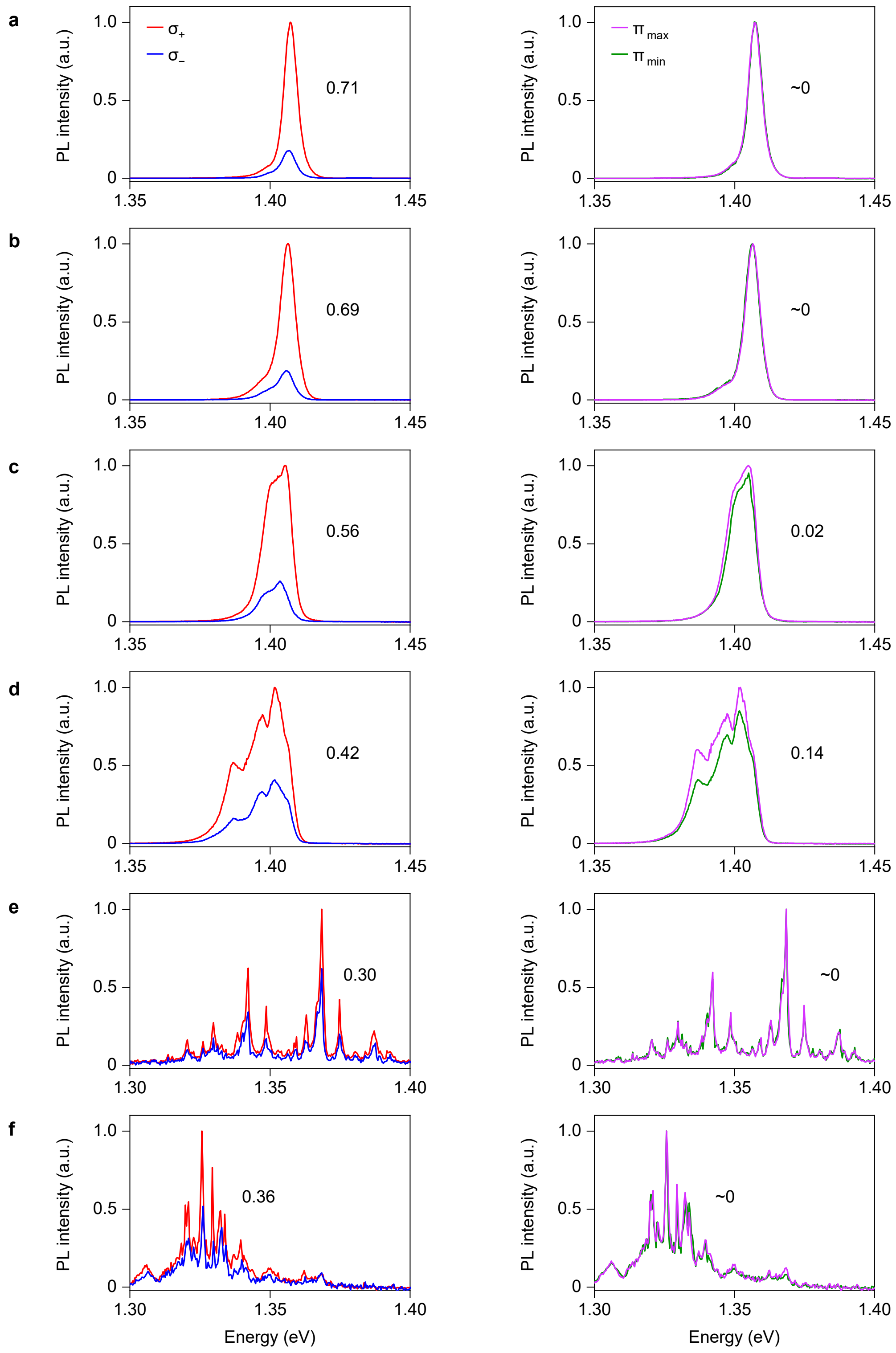

Supplementary Figure 23. **a** – **f**, PL spectra recorded in circular (left panels, with σ_+ and σ_- polarized detection shown in red and blue, respectively) and linear (right panels, with two orthogonal orientations of linear polarization for maximum and minimum PL intensity shown in purple and green, respectively) basis from the same regions as in Supplementary Fig. 22 **a** – **f**. The numbers in each panel denote the respective degrees of circular and linear polarization, P_c and P_l.

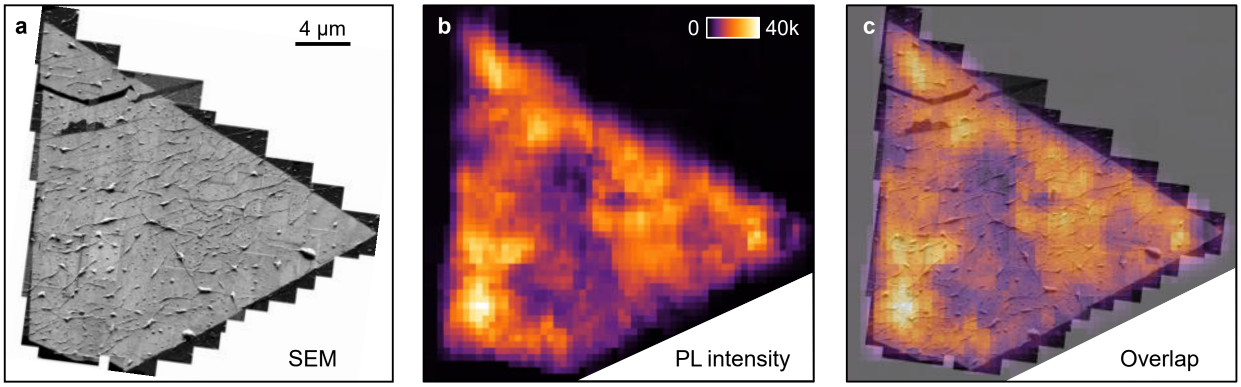

Supplementary Figure 24. Same as Supplementary Fig. 21 but for the R-type sample.

In the same manner, we performed secondary electron modulated SEM and PL hyperspectral imaging on the R-type sample and co-aligned the two images (Supplementary Fig. 24). From the direct correlation between SEM and PL images, we again obtained the results consistent with the analysis in the main text on reconstruction patterns and their optical characteristics of R-type samples. For 2D domains of R_h^X stacking, exemplified in Supplementary Fig. 25 **a** and **b**, their interlayer exciton PL is bright and the spectra feature a single peak at $\sim$1.33 eV from the singlet exciton state. Consistently, their DR spectra show single-peak resonances of intralayer excitons in $MoSe_2$ and WSe_2. The interlayer exciton PL from these 2D domains has negative degree of circular polarization and zero degree of linear polarization (Supplementary Fig. 26 **a** and **b**). For 1D stripe regions, exemplified in Supplementary Fig. 25 **c** and **d**, the interlayer exciton PL is blue-shifted and broadened compared to that from 2D domains of R_h^X stacking. This PL features almost zero degree of circular polarization but high degree of linear polarization along the elongated direction of the stripes. Finally, for 0D array regions, exemplified in Supplementary Fig. 25 **e** and **f**, the interlayer exciton PL is reduced and blue-shifted by several tens of meV compared to 2D domains. In contrast to H-type, the smaller 0D domains show larger blue-shifts because of the quantum confinement. When excitation powers are below 100 nW, the PL spectra of 0D domains have the characteristic of quantum dots, showing narrow peaks with negative degree of circular polarization and zero degree of linear polarization (Supplementary Fig. 26 **e** and **f**). The DR spectra of 0D regions in R-type exhibit for both $MoSe_2$ and WSe_2 intralayer exciton resonances characteristic splittings and broadenings with smaller domains featuring larger energy splittings, which is similar to H-type.

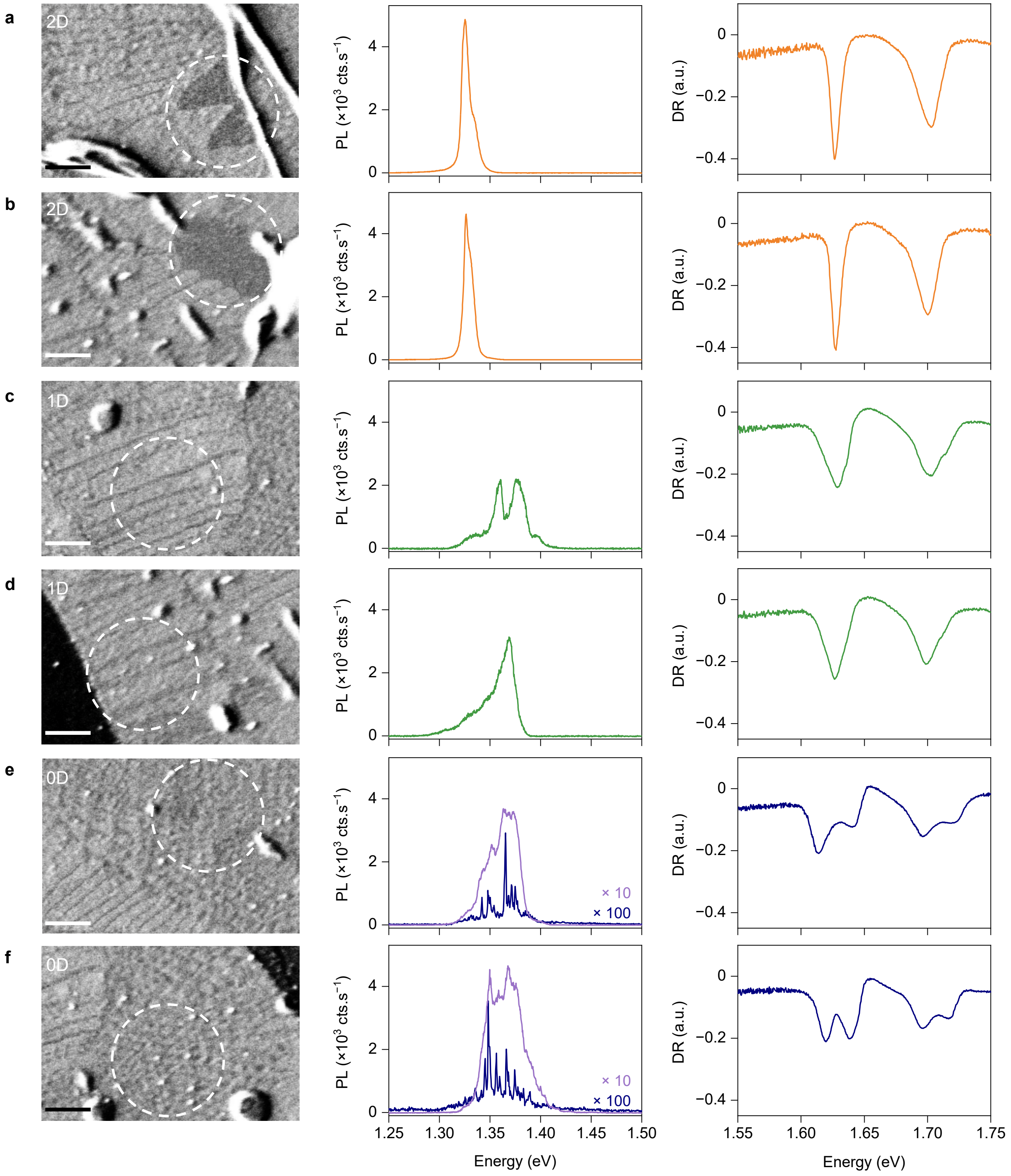

Supplementary Figure 25. Examples of reconstruction patterns and the corresponding optical spectra in the R-type sample. **a** – **f**, Left panels show representative areas of 2D (**a** and **b**), 1D (**c** and **d**) and 0D (**e** and **f**) domains identified in SEM imaging (the scale bars are 250 nm). Central and right panels show the corresponding PL and DR spectra within the optical spot delimited by dashed circles. The PL spectra were excited with 2 μW laser power. For 0D domains, the PL spectra at 0.01 μW are shown (dark purple) with respective scaling factors in addition to the spectra recorded at 2 μW (light purple).

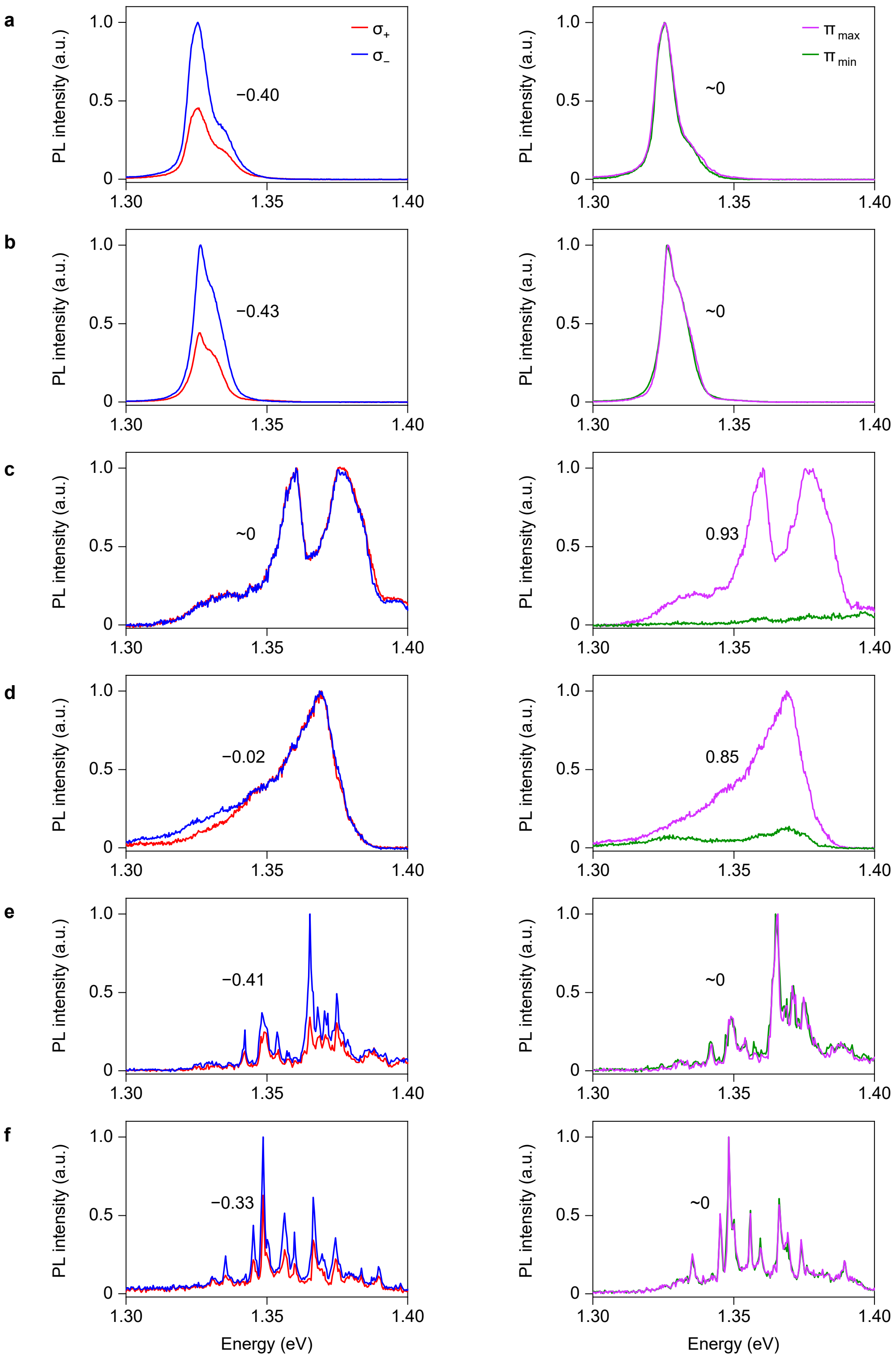

Supplementary Figure 26. **a** – **f**, PL spectra recorded in circular (left panels, with σ_+ and σ_- polarized detection shown in red and blue, respectively) and linear (right panels, with two orthogonal orientations of linear polarization for maximum and minimum PL intensity shown in purple and green, respectively) basis from the same regions as in Supplementary Fig. 25 **a** – **f**. The numbers in each panel denote the respective degrees of circular and linear polarization, P_c and P_l.

Supplementary Note 6: Analysis of intralayer exciton absorption spectra in reconstructed domains

In the following, we analyze the DR spectra of intralayer excitons in Fig. 3a and 4a (main text) recorded on different positions of reconstructed R- and H-stacks. First, we note that the DR spectra exhibit asymmetric lineshapes due to multiple interferences at the interfaces of samples containing stacks of hBN, TMD, SiO_2 and Si. To simplify the lineshape analysis, we transform the DR spectra into absorption spectra by adapting the numerical method developed in Ref. [6].

We begin with a simple case of two interfaces provided by a thin TMD layer on top of SiO_2 in vacuum. Since the thickness of TMD layer d is much smaller than the wavelength of light λ, DR (defined as ΔR/R) can be written as [7]:

$$\mathrm{DR} = -\frac{8\pi d}{\lambda}\,\mathrm{Im}\left(\frac{\epsilon_1-\epsilon_2}{\epsilon_1-\epsilon_3}\right), \tag{12}$$

where $\epsilon_{1,2,3}$ is the dielectric function of vacuum, TMD layer and SiO_2. In linear response approximation, $\epsilon_{1,2,3} = 1 + \chi_{1,2,3}$ with dielectric susceptibility of each layer $\chi_{1,2,3}$. With vanishing absorption in vacuum and SiO_2 at the wavelengths of relevance in the spectral window of intralayer exciton transitions in $MoSe_2$ and WSe_2, $\mathrm{Im}(\epsilon_1) = \mathrm{Im}(\epsilon_3) = 0$, and thus DR in Eq. 12 is simply proportional to the imaginary part of the optical susceptibility χ_2'' of the thin TMD layer, which in turn corresponds to its absorption.

To model the DR response of our samples with two hBN layers that embed the TMD layer on the top and bottom sides and ~ 500 μm Si substrate underneath the SiO_2 layer, we account for additional interfaces between the top hBN layer and the TMD layer, the TMD layer and the bottom hBN layer, its interface to SiO_2, and the SiO_2-Si interface by a phase factor $e^{i\alpha}$ [8] in the effective susceptibility $\tilde{\chi}_2 = e^{-i\alpha_0}\chi_2$, where we approximate α by a constant α_0 in the relevant wavelength range. By decomposing $\tilde{\chi}_2$ into real and imaginary parts as $\tilde{\chi}_2' + i\tilde{\chi}_2''$, the imaginary part of the effective susceptibility $\tilde{\chi}_2''$ can thus be expressed as $\chi_2'' = \cos(\alpha_0)\tilde{\chi}_2'' + \sin(\alpha_0)\tilde{\chi}_2'$. Finally, as DR is proportional to $\tilde{\chi}_2''$, using Kramers-Kronig relations we obtain:

$$\begin{aligned}\chi_2''(\omega) &= \cos(\alpha_0)\;\tilde{\chi}_2''(\omega) + \sin(\alpha_0)\,\tfrac{2}{\pi}\mathcal{P}\textstyle\int_0^\infty \frac{\omega'\tilde{\chi}_2''(\omega')}{\omega'^2-\omega^2}d\omega' \\ &\propto \cos(\alpha_0)\mathrm{DR}(\omega) + \sin(\alpha_0)\,\tfrac{2}{\pi}\mathcal{P}\textstyle\int_0^\infty \frac{\omega'\mathrm{DR}(\omega')}{\omega'^2-\omega^2}d\omega',\end{aligned} \tag{13}$$

where ω denotes the angular frequency. Using Eq. 13, we compute $\chi_2''(\omega)$ of the exciton transitions in the regions of $MoSe_2$ and WSe_2 monolayers from the respective DR spectra

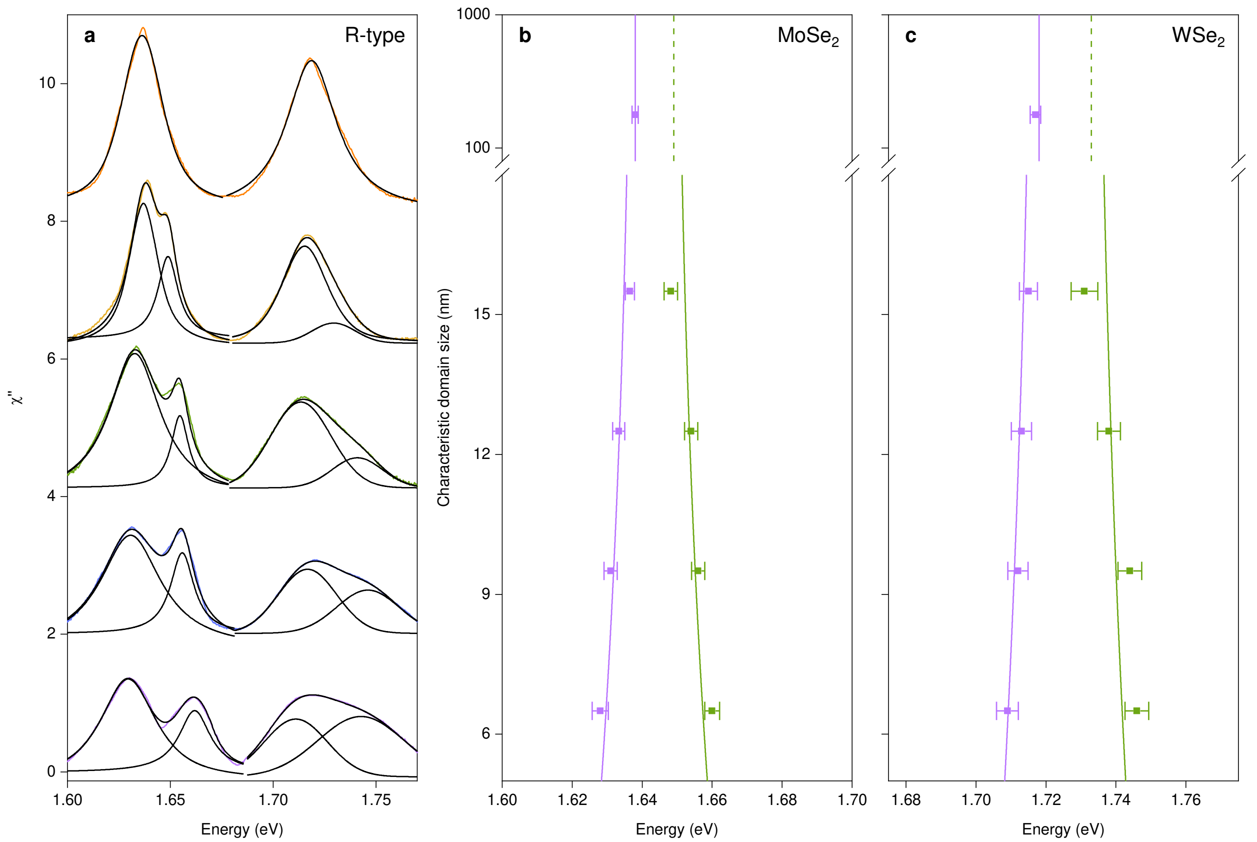

Supplementary Figure 27. **a**, Absorption χ'' spectra (colored lines) of $MoSe_2$ and WSe_2 intralayer excitons upon gradual displacement from a bright to a dark region in R-type HBL (shown from top to bottom), obtained from the DR spectra of Fig. 3a of the main text and fitted to multiple peaks with a Voigt profile (black lines). The top-most spectrum exhibits single-peak resonances in large 2D domains; the spectra below feature two-peak resonances with a splitting sensitive to the size of reconstructed domains. **b** and **c**, Model analysis (solid lines) of the peak splitting in the intralayer exciton transition doublets of $MoSe_2$ and WSe_2, respectively, as a function of the characteristic size of reconstructed domains. The data points (dots) and their error bars were extracted from best multiple-peak fits to the corresponding spectra in **a**.

in the charge-neutral regime for different values of the phase shift α_0 from $-\pi$ to π. Since the exciton absorption of monolayer TMD is expected to have a Lorentzian lineshape in the absence of free charge carriers [9], we choose the value of α_0 such that the obtained absorption spectra $\chi_2''(\omega)$ can be accurately fitted by a Lorentzian. Successively, we use this value of α_0 to compute the absorption spectra of intralayer excitons in HBL regions as the transition energies are similar to monolayer exciton transitions. The respectively

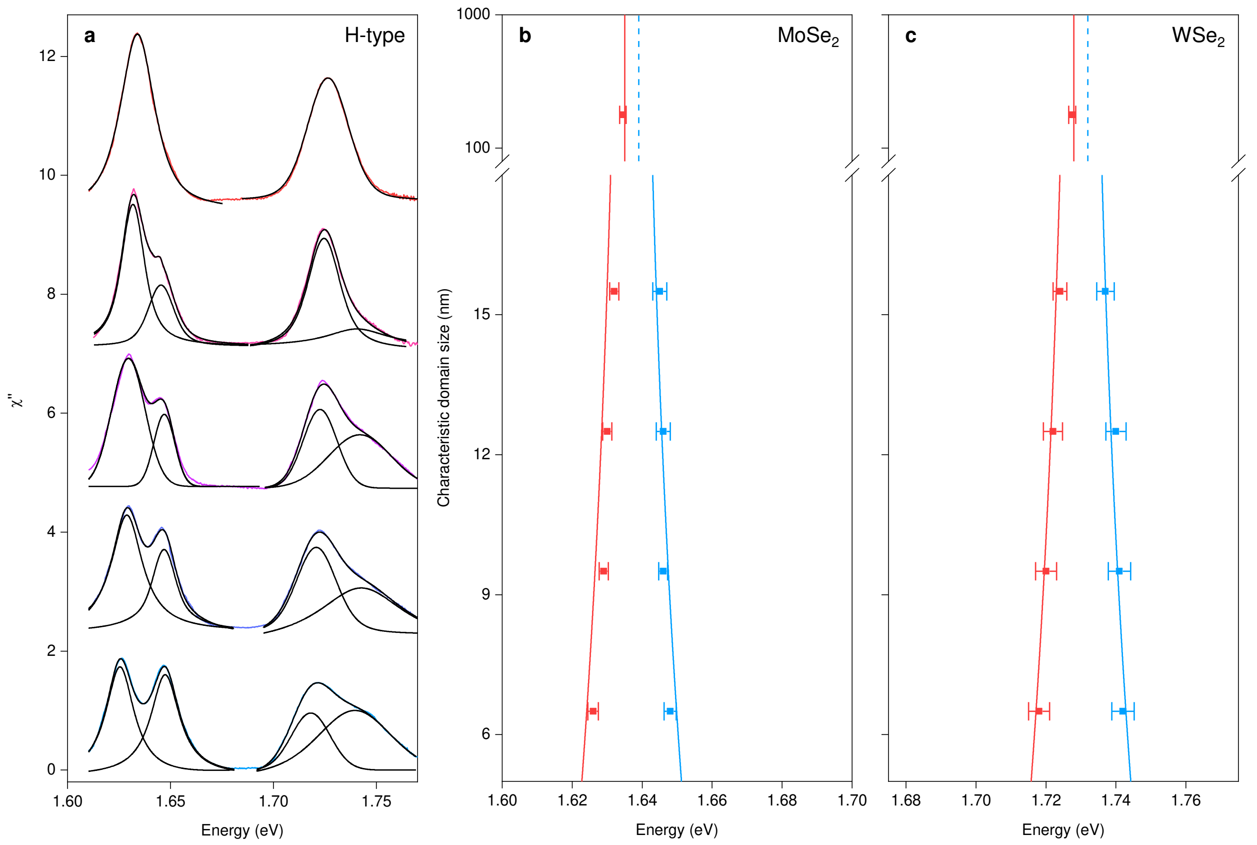

Supplementary Figure 28. **a**, Absorption χ'' spectra (colored lines) of $MoSe_2$ and WSe_2 intralayer excitons upon gradual displacement from a bright to a dark region in H-type HBL (shown from top to bottom), obtained from the DR spectra of Fig. 4a of the main text and fitted to multiple peaks with a Voigt profile (black lines). The top-most spectrum exhibits single-peak resonances in large 2D domains; the spectra below feature two-peak resonances with a splitting sensitive to the size of reconstructed domains. **b** and **c**, Model analysis (solid lines) of the peak splitting in the intralayer exciton transition doublets of $MoSe_2$ and WSe_2, respectively, as a function of the characteristic size of reconstructed domains. The data points (dots) and their error bars were extracted from best multiple-peak fits to the corresponding spectra in **a**.

obtained absorption spectra $\chi_2''(\omega)$ in Supplementary Fig. 27 and 28 are predominantly positive, providing confidence in our approach.

In the following analysis of absorption spectra, we assume intralayer exciton transition energies are sensitive to the local stacking and domain size. In regions of two proximal stackings A and B (R_h^X and R_h^M, and H_h^h and H_h^X in the case of 0D reconstructed domains of R- and H-stacks, respectively) with the corresponding energy minima, coupling of the

intralayer exciton states via tunneling gives rise to a doublet peak structure. By introducing tunnel-hopping between the domains of different registries, we model the eigenenergies of the coupled two-level system with the following Hamiltonian:

$$H = \begin{pmatrix} E_A & te^{-\beta a_m} \\ te^{-\beta a_m} & E_B \end{pmatrix}, \quad (14)$$

where E_A and E_B are the intralayer exciton energies for the respective commensurate stackings, t and β are the hopping parameters, and a_m is the domain size (or moiré period). Using $t = 20$ meV and $\beta = 0.07$ nm^{-1} estimated from the calculations of Yu et al. [10], we compute the energies (in meV) in Supplementary Tab. 2 by fitting the peak evolution in Supplementary Fig. 27 and 28.

	R, $MoSe_2$	R, WSe_2	H, $MoSe_2$	H, WSe_2
E_A	1638	1718	1635	1728
E_B	1649	1733	1639	1732

Supplementary Table 2. Energies (in meV) of intralayer excitons derived from absorption spectra analysis in the framework of a tunnel-coupled two-level system. E_A and E_B refer to the interlayer excitons energies in R_h^X and R_h^M as well as in H_h^h and H_h^X registries of R- and H-stacks, respectively.

Supplementary Note 7: Density functional theory calculations

We used density functional theory (DFT) to calculate exciton g-factors and oscillator strengths of interlayer excitons in R- and H-type $MoSe_2$-WSe_2 HBLs as described in detail in Refs. [11, 12]. DFT calculations were performed with the PBEsol exchange-correlation functional [13] as implemented in the Vienna ab- initio simulation package (VASP) [14]. Van der Waals interactions were included with the DFT-D3 method [15] and Becke-Johnson damping [16]. Moreover, spin-orbit interactions were included at all stages. Elementary cells with thickness of 35 Å in the z-direction were used to minimize the interactions between periodic images. The atomic positions were relaxed with a cutoff energy of 400 eV until the total energy change was less than 10^{-6} eV. Calculations were performed for high-symmetry points of HBL moiré patterns in R- and H-type stackings on the Γ-centered $\mathbf{k}$ grid of 6×6 divisions with 600 bands and the cutoff energy of 300 eV.

The theoretical g-factors and oscillator strengths of interlayer excitons obtained from our DFT calculations for R- and H-type $MoSe_2$-WSe_2 HBLs are listed in Table. 1 of the main

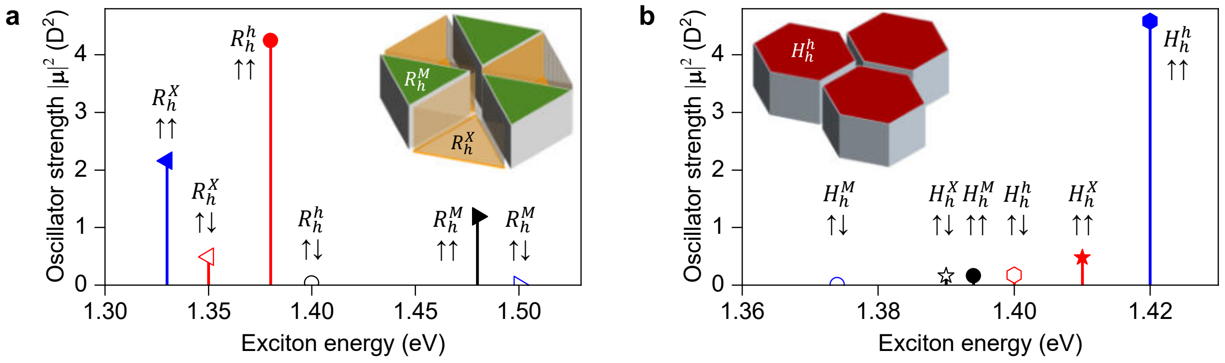

Supplementary Figure 29. **a** and **b**, Oscillator strengths of interlayer exctons in R- and H-type $MoSe_2$-WSe_2 HBLs. The dipolar selection rules are represented by colors (red: σ_+; blue: σ_-; black: z-polarized in-plane emission), filled and empty symbols indicate the corresponding singlet and triplet exciton states. The inset schematics show the energy landscape in the presence of periodic reconstruction, with excitons in triangular R_h^X domains confined by the potential walls of R_h^M domains, and hexagonal H_h^h domains with highest-energy excitons.

text. For completeness, we list in Supplementary Table 3 interlayer exciton g-factors in different stackings and momentum-dark spin-valley configurations. Supplementary Fig. 29 gives a graphical representation of the energetic ordering of singlet and triplet interlayer exciton states with respective dipolar selection rules and oscillator strengths, labelled by the according spin configuration and atomic registry of high symmetry stackings in R- and H-type HBLs. The insets of Supplementary Fig. 29 a and b show the exciton energy landscape in R- and H-type stacking in the presence of periodic reconstruction, with excitons in triangular R_h^X domains confined by the surrounding potential walls of R_h^M domains, and domains of highest-energy H_h^h excitons in hexagonal tiling. Note that in such potential landscapes, the energy of R_h^X excitons will increase in nanosized domains due to quantum confinement, whereas a decrease is expected for H_h^h excitons due to decreasing potential energy in the surrounding domains. This distinction explains both the blue-shift of quantum dot type peaks for 0D arrays with respect to the R_h^X singlet emission in R-type 2D domains and the red-shift of H_h^h quantum dot features below the emission peak of H-type 2D domains.

Exciton	Spin configuration	R-type R_h^X	R-type R_h^h	R-type R_h^M	H-type H_h^M	H-type H_h^X	H-type H_h^h
$K'K$ (KK)	singlet	13.1	13.0	13.0	6.5	6.4	5.7
$K'K$ (KK)	triplet	17.8	17.6	17.6	11.1	11.0	10.4
QK	singlet	8.6	9.0	8.7	10.4	10.0	9.6
QK	triplet	13.0	13.3	12.9	13.9	13.6	13.2
$Q'K$	singlet	10.6	10.7	11.0	10.3	10.5	10.0
$Q'K$	triplet	14.9	15.0	15.3	13.8	14.2	13.7
$K\Gamma$	singlet	4.0	3.6	3.7	3.0	3.1	3.3
$K\Gamma$	triplet	0.7	1.0	1.0	7.6	7.6	7.9
$K'\Gamma$	singlet	3.3	3.0	3.0	3.6	3.6	3.9
$K'\Gamma$	triplet	8.0	7.6	7.7	1.0	0.9	0.7
$Q\Gamma$	singlet	1.1	1.0	1.3	0.3	0.1	0.0
$Q\Gamma$	triplet	3.2	3.3	2.9	3.8	3.5	3.6
$Q'\Gamma$	singlet	0.8	0.7	1.1	0.2	0.5	0.4
$Q'\Gamma$	triplet	5.1	5.0	5.3	3.7	4.1	4.0

Supplementary Table 3. Landé g-factor values of momentum-indirect interlayer excitons in R- and H-type $MoSe_2$-WSe_2 HBL calculated from DFT. We restrict the table to lowest-energy interlayer exciton states formed between conduction band electrons in K, K', Q or Q' valleys of $MoSe_2$ and valence band holes at K in WSe_2 or at Γ in the hybrid band of $MoSe_2$-WSe_2 according to the electronic band structure [17]. For each state, the absolute g-factor values of both singlet and triplet spin configurations are listed for distinct atomic registries. Note that due to different symmetry in R- and H-type $MoSe_2$-WSe_2 HBLs, the spin-valley configurations are distinct [18]: In R-type (H-type), KK $(K'K)$ interlayer exciton states are momentum-direct with lowest energy states in singlet (triplet) configuration, whereas $K'K$ (KK) states are momentum-indirect with triplet (singlet) lowest-energy states.

Supplementary References

[1] K. Ashida, T. Kajino, Y. Kutsuma, N. Ohtani, and T. Kaneko, Crystallographic orientation dependence of sem contrast revealed by SiC polytypes, J. Vac. Sci. Technol. B **33**, 04E104 (2015).

[2] T. I. Andersen, G. Scuri, A. Sushko, K. De Greve, J. Sung, Y. Zhou, D. S. Wild, R. J. Gelly, H. Heo, D. Bérubé, A. Y. Joe, L. A. Jauregui, K. Watanabe, T. Taniguchi, P. Kim, H. Park, and M. D. Lukin, Excitons in a reconstructed moiré potential in twisted WSe_2/WSe_2 homobilayers, Nat. Mater. **20**, 480 (2021).

[3] V. V. Enaldiev, V. Zólyomi, C. Yelgel, S. J. Magorrian, and V. I. Fal'ko, Stacking domains and dislocation networks in marginally twisted bilayers of transition metal dichalcogenides, Phys. Rev. Lett. **124**, 206101 (2020).

[4] S. Carr, D. Massatt, S. B. Torrisi, P. Cazeaux, M. Luskin, and E. Kaxiras, Relaxation and domain formation in incommensurate two-dimensional heterostructures, Phys. Rev. B **98**, 224102 (2018).

[5] V. V. Enaldiev, F. Ferreira, S. J. Magorrian, and V. I. Fal'ko, Piezoelectric networks and ferroelectric domains in twistronic superlattices in WS_2/MoS_2 and $WSe_2/MoSe_2$ bilayers, 2D Mater. **8**, 025030 (2021).

[6] P. Back, M. Sidler, O. Cotlet, A. Srivastava, N. Takemura, M. Kroner, and A. Imamoğlu, Giant paramagnetism-induced valley polarization of electrons in charge-tunable monolayer $mose_2$, Phys. Rev. Lett. **118**, 237404 (2017).

[7] J. McIntyre and D. Aspnes, Differential reflection spectroscopy of very thin surface films, Surface Science **24**, 417 (1971).

[8] A. Arora, A. Mandal, S. Chakrabarti, and S. Ghosh, Magneto-optical Kerr effect spectroscopy based study of Landé g-factor for holes in GaAs/AlGaAs single quantum wells under low magnetic fields, J. Appl. Phys. **113**, 213505 (2013).

[9] G. Scuri, Y. Zhou, A. A. High, D. S. Wild, C. Shu, K. De Greve, L. A. Jauregui, T. Taniguchi, K. Watanabe, P. Kim, M. D. Lukin, and H. Park, Large excitonic reflectivity of monolayer $mose_2$ encapsulated in hexagonal boron nitride, Phys. Rev. Lett. **120**, 037402 (2018).

[10] H. Yu, G.-B. Liu, J. Tang, X. Xu, and W. Yao, Moiré excitons: From programmable quantum emitter arrays to spin-orbit-coupled artificial lattices, Sci. Adv. **3**, e1701696 (2017).

[11] J. Förste, N. V. Tepliakov, S. Yu. Kruchinin, J. Lindlau, V. Funk, M. Förg, K. Watanabe, T. Taniguchi, A. S. Baimuratov, and A. Högele, Exciton g-factors in monolayer and bilayer WSe_2 from experiment and theory, Nat. Commun. **11**, 4539 (2020).

[12] M. Förg, A. S. Baimuratov, S. Yu. Kruchinin, I. A. Vovk, J. Scherzer, J. Förste, V. Funk, K. Watanabe, T. Taniguchi, and A. Högele, Moiré excitons in $MoSe_2/WSe_2$ heterobilayers and heterotrilayers, Nat. Commun. **12**, 1656 (2021).

[13] G. I. Csonka, J. P. Perdew, A. Ruzsinszky, P. H. T. Philipsen, S. Lebègue, J. Paier, O. A. Vydrov, and J. G. Ángyán, Assessing the performance of recent density functionals for bulk solids, Phys. Rev. B **79**, 155107 (2009).

[14] G. Kresse and J. Furthmüller, Efficient iterative schemes for ab initio total-energy calculations using a plane-wave basis set, Phys. Rev. B **54**, 11169 (1996).

[15] S. Grimme, J. Antony, S. Ehrlich, and H. Krieg, A consistent and accurate ab initio parametrization of density functional dispersion correction (DFT-D) for the 94 elements H-Pu, J. Chem. Phys. **132**, 154104 (2010).

[16] S. Grimme, S. Ehrlich, and L. Goerigk, Effect of the damping function in dispersion corrected density functional theory, J. Comp. Chem. **32**, 1456 (2011).

[17] R. Gillen and J. Maultzsch, Interlayer excitons in $MoSe_2/WSe_2$ heterostructures from first principles, Phys. Rev. B **97**, 165306 (2018).

[18] M. Förg, L. Colombier, R. K. Patel, J. Lindlau, A. D. Mohite, H. Yamaguchi, M. M. Glazov, D. Hunger, and A. Högele, Cavity-control of interlayer excitons in van der Waals heterostructures, Nat. Commun. **10**, 1 (2019).
